# Disruption of miR-29 Leads to Aberrant Differentiation of Smooth Muscle Cells Selectively Associated with Distal Lung Vasculature

**DOI:** 10.1371/journal.pgen.1005238

**Published:** 2015-05-28

**Authors:** Leah Cushing, Stefan Costinean, Wei Xu, Zhihua Jiang, Lindsey Madden, Pingping Kuang, Jingshu Huang, Alexandra Weisman, Akiko Hata, Carlo M. Croce, Jining Lü

**Affiliations:** 1 Boston University School of Medicine, Boston, Massachusetts, United States of America; 2 Department of Pathology, Ohio State Wexner Medical Center, The Ohio State University, Columbus, Ohio, United States of America; 3 Columbia Center for Human Development, Division of Pulmonary, Allergy & Critical Care Medicine, Department of Medicine, Columbia University, College of Physicians & Surgeons, New York, New York, United States of America; 4 Molecular Cardiology Research Institute, Tufts Medical Center, Boston, Massachusetts, United States of America; 5 Cardiovascular Research Institute, University of California, San Francisco, San Francisco, California, United States of America; 6 Department of Molecular Virology, Immunology and Medical Genetics, The Ohio State University, Columbus, Ohio, United States of America; University of California Berkeley, UNITED STATES

## Abstract

Differentiation of lung vascular smooth muscle cells (vSMCs) is tightly regulated during development or in response to challenges in a vessel specific manner. Aberrant vSMCs specifically associated with distal pulmonary arteries have been implicated in the pathogenesis of respiratory diseases, such as pulmonary arterial hypertension (PAH), a progressive and fatal disease, with no effective treatment. Therefore, it is highly relevant to understand the underlying mechanisms of lung vSMC differentiation. miRNAs are known to play critical roles in vSMC maturation and function of systemic vessels; however, little is known regarding the role of miRNAs in lung vSMCs. Here, we report that miR-29 family members are the most abundant miRNAs in adult mouse lungs. Moreover, high levels of miR-29 expression are selectively associated with vSMCs of distal vessels in both mouse and human lungs. Furthermore, we have shown that disruption of miR-29 *in vivo* leads to immature/synthetic vSMC phenotype specifically associated with distal lung vasculature, at least partially due to the derepression of KLF4, components of the PDGF pathway and ECM-related genes associated with synthetic phenotype. Moreover, we found that expression of FBXO32 in vSMCs is significantly upregulated in the distal vasculature of miR-29 null lungs. This indicates a potential important role of miR-29 in smooth muscle cell function by regulating FBXO32 and SMC protein degradation. These results are strongly supported by findings of a cell autonomous role of endogenous miR-29 in promoting SMC differentiation *in vitro*. Together, our findings suggested a vessel specific role of miR-29 in vSMC differentiation and function by targeting several key negative regulators.

## Author Summary

The pathogenesis of some vascular diseases, such as PAH is selectively associated with aberrant differentiation and proliferation of vSMCs of distal arteries. While significant progresses have been made in understanding the core mechanism of differentiation and proliferation of vSMCs, little is known regarding vessel specific regulations. By investigating the expression and function of miR-29 *in vivo*, we found a vessel selective enriched expression and function of miR-29 during mouse lung development. Interestingly, disruption of miR-29 results in defects in vSMCs differentiation of distal vessels, reminiscent of vSMC phenotype observed in the early stage of PAH in which immature/synthetic vSMCs of distal arteries failed to differentiate and were unable to tune down the expression of collagens and other extracellular-related genes. This is the first evidence that miR-29 selectively regulates vSMCs differentiation and vessel wall formation. Future implications are to study the expression and function of miR-29 in human pulmonary vascular diseases, which might lead to establishing miR-29 as a therapeutic target for disease intervention.

## Introduction

The differentiation of lung vSMCs along the longitudinal axis from the hilum of the lung to the most peripheral vessels during development is tightly regulated for proper formation and maturation of vessel walls. While most vSMCs of distal vessels are differentiated from surrounding mesenchymal cells in a process of vasculogenesis, the origin of proximal vSMCs might be more complex and include the contribution of airway SMCs[1–3]. In general, vSMCs of proximal vessels are more differentiated than those of distal vasculature during development. In human and other animal models examined, distal lung vSMCs adopt an immature/synthetic phenotype associated with active proliferation, migration and ECM production during embryonic and early postnatal development [3,4]. They then gradually switch to a mature/contractile phenotype with a significant upregulation of contractile and cytoskeletal proteins, together with the diminished proliferation and reduced ECM production [3–5].

Understanding mechanisms of lung vSMC differentiation is highly relevant, since aberrant vSMC proliferation and differentiation have been implicated in the pathogenesis of lung vascular diseases, such as pulmonary arterial hypertension (PAH), a progressive and fatal disease without effective treatment[6–9]. vSMCs from different organs or structures share core regulatory networks for their proliferation and differentiation. Results from extensive studies have shown that key transcriptional regulators including serum response factor (Srf), myocardin (Myocd), myocardin-related transcription factors (Mrtfs) and members of krüppel-like transcription factor (Klf4/Klf5), play instrumental roles in the proliferation and differentiation of vSMCs [2,10,11]. vSMC proliferation and differentiation is also significantly influenced by the activity of PDGF signaling [12,13]. Pulmonary vSMCs carrying mutation of *bone morphogenetic protein type-2 receptor* (*BMPR2*), which accounts for 70% of the heritable form of PAH, also predisposes vSMC to switch to a more synthetic phenotype, resulting in reduced expression of contractile proteins and increased proliferation and migration[14–16]. vSMC phenotype is also influenced by distinct local environmental signal and gene network during development. vSMCs of different vessels differ in their expression of ion channels, hormone receptors and components of signaling pathways. Despite these insights, still little is known regarding the mechanisms regulating lung vSMC differentiation during development.

miRNAs are small non-coding RNAs important for posttranscriptional regulation of gene expression [17]. Examination of systemic vessels, such as the aorta, showed the critical roles of miRNAs in vSMC differentiation and vessel wall development [18–20]. However, little is known about miRNAs in pulmonary vSMC differentiation. Here, we report a vessel specific role of miR-29 in promoting the differentiation of vSMC during mouse lung development. We found that miR-29 family members are abundantly, selectively and dynamically expressed during mouse and human lung development. We showed that miR-29 promotes vSMCs differentiation by targeting Klf4, the PDGF pathway and ECM-related synthetic markers. Moreover, disruption of miR-29 leads to significant upregulation of Foxo3a/Fbxo32, a pathway well known for skeletal muscle wasting, which might lead to excessive degradation of important smooth muscle cell proteins.

## Results

### Expression of miR-29 in vasculature of mouse lungs

**Fig 1:**
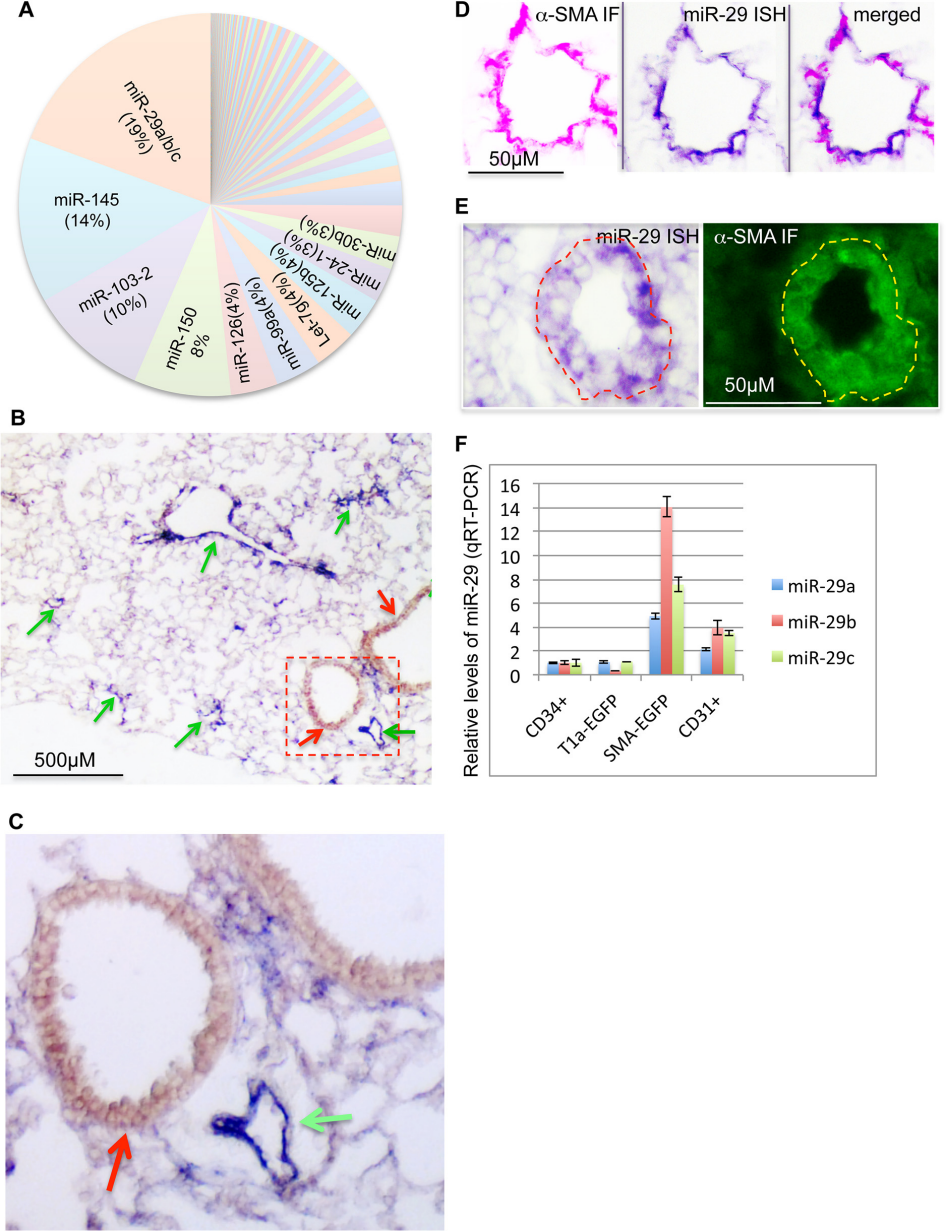
Expression of miR-29 in mouse lungs. **(A)** miR-29 family members are the most abundant miRNAs in adult mouse lungs, representing about 19% of total reads of known miRNAs (Next Generation of Sequencing). **(B)** High levels of miR-29 ISH signal (purple, miR-29c probe, miR-29a or miR-29b probes revealed the same pattern) are associated with vasculature (green arrows), miR-29 in SMCs underneath airway epithelium is much lower (red arrow). **(C)** Enlarged image of outlined area of **B**. **(D)** miR-29 ISH signal (purple) co-localizes with α-SMA IF signal (red) in vessel walls of distal lungs. **(E)** High levels of miR-29 (purple) in α-SMA positive cells (green) of vessel walls of E18.5 lungs. **(F)** Levels of miR-29a/b/c in isolated SMCs or endothelial cells (CD31 positive) of adult lungs are significantly higher than those of CD34 positive or Type I epithelial cells (T1α-GFP positive)(P<0.05, Student’s *t* test). Relative levels were calculated by comparing with levels of miR-29a/b/c in CD34 positive cells (n = 3); Data are mean ± SEM.

To characterize the genome-wide expression profile of miRNAs, we sequenced small RNA (>40nt) samples isolated from lungs in different developmental stages or adult mice using Next Generation of Sequencing (NGS) (ABI, SOLiD system). This profiling revealed that miR-29 family members (miR-29a/b/c) transcribed from two genomic loci, *miR-29a/b1* and *miR-29b2/c*, are the most abundant miRNAs in adult mouse lungs (Fig 1A). The total number of reads for miR-29a/b/c is 190,767 per million, representing about 19% of total known miRNAs of adult lungs.

**Fig 2:**
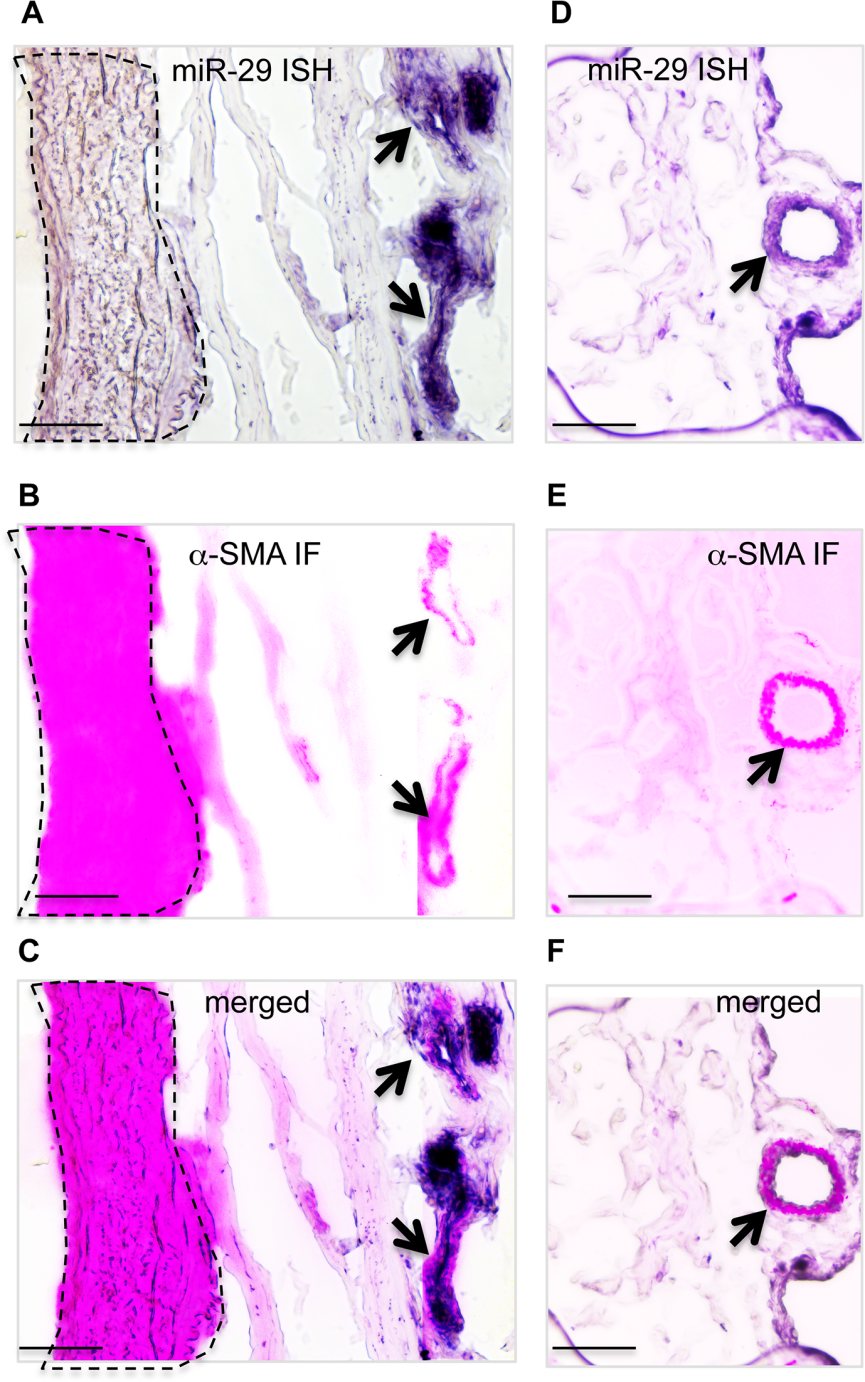
Expression of miR-29 in human lung vasculature. **(A, B&C)** High miR-29c ISH signal (purple) co-localizes with α-SMA IF staining signal (red) of small vessels (black arrow), while the level of miR-29c in vSMCs (media layer) of nearby large pulmonary arteries (highlighted area by dashed lines) is much lower. **(D, E&F)** miR-29 is also highly expressed in vSMCs of distal pulmonary artery, where it co-localizes with α-SMA IF staining signal (black arrow). Scale bar: 50μM.

To examine the expression pattern, we performed in situ hybridization (ISH) for miR-29 using DIG-labeled LNA probes. The strongest ISH signal of miR-29 in adult mouse lungs was detected in distal vascular structures (Fig 1B and 1C). By co-staining with α-SMA, we found that miR-29 ISH signal co-localizes with α-SMA within vessel walls suggesting an enriched expression in vSMCs (Fig 1D). In embryonic day 18.5 (E18.5) lungs, in which α-SMA positive cells are found in limited numbers of distal vessels with relatively thick walls, a typical morphological feature before extrauterine adaptation; high levels of miR-29 were also selectively detected in α-SMA positive cells of these vessel walls (Fig 1E). Interestingly, levels of miR-29 in the media layer of large arteries, such as the dorsal aorta, where vSMCs reside, are much lower (S1A and S1B Fig). Moreover, levels of miR-29 in airway SMCs is also much lower than those present in the distal vascular structure (Fig 1B and 1C). To further examine miR-29 expression in SMCs, we sorted and collected SMCs (α-SMA-EGFP transgenic mice) or type I epithelial cells (T1α-EGFP transgenic mice, gift from Dr. Maria Ramirez, Boston University Medical Center) or hematopoietic cells (live cell staining of CD34) or endothelial cells (live cell CD31 staining) from adult mouse lungs[21]. By qRT-PCR, we found that levels of miR-29a/b/c in α-SMA-EGFP positive cells are the highest, with four to fourteen folds higher than those of CD34 positive cells (Fig 1F). Significant levels of miR-29a/b/c are also detected in endothelial cells (CD31 positive) (Fig 1F). We then examined whether this vessel specific pattern is also present in human lungs, and found that high levels of miR-29 is selectively detected in vSMCs of small arteries including pulmonary arteries, but not in the vSMCs (media layer) of large pulmonary arteries (Fig 2A, 2B and 2C). In distal small arteries, miR-29 specifically co-localizes with α-SMA staining (Fig 2D, 2E and 2F). Together our ISH analysis revealed a vessel specific expression of miR-29 in vSMCs in both human and mouse lungs, suggesting a conserved expression pattern in vSMCs of distal vessels including those of distal pulmonary arteries.

### Disruption of miR-29 expression *in vivo* results in aberrant vSMC differentiation

**Fig 3:**
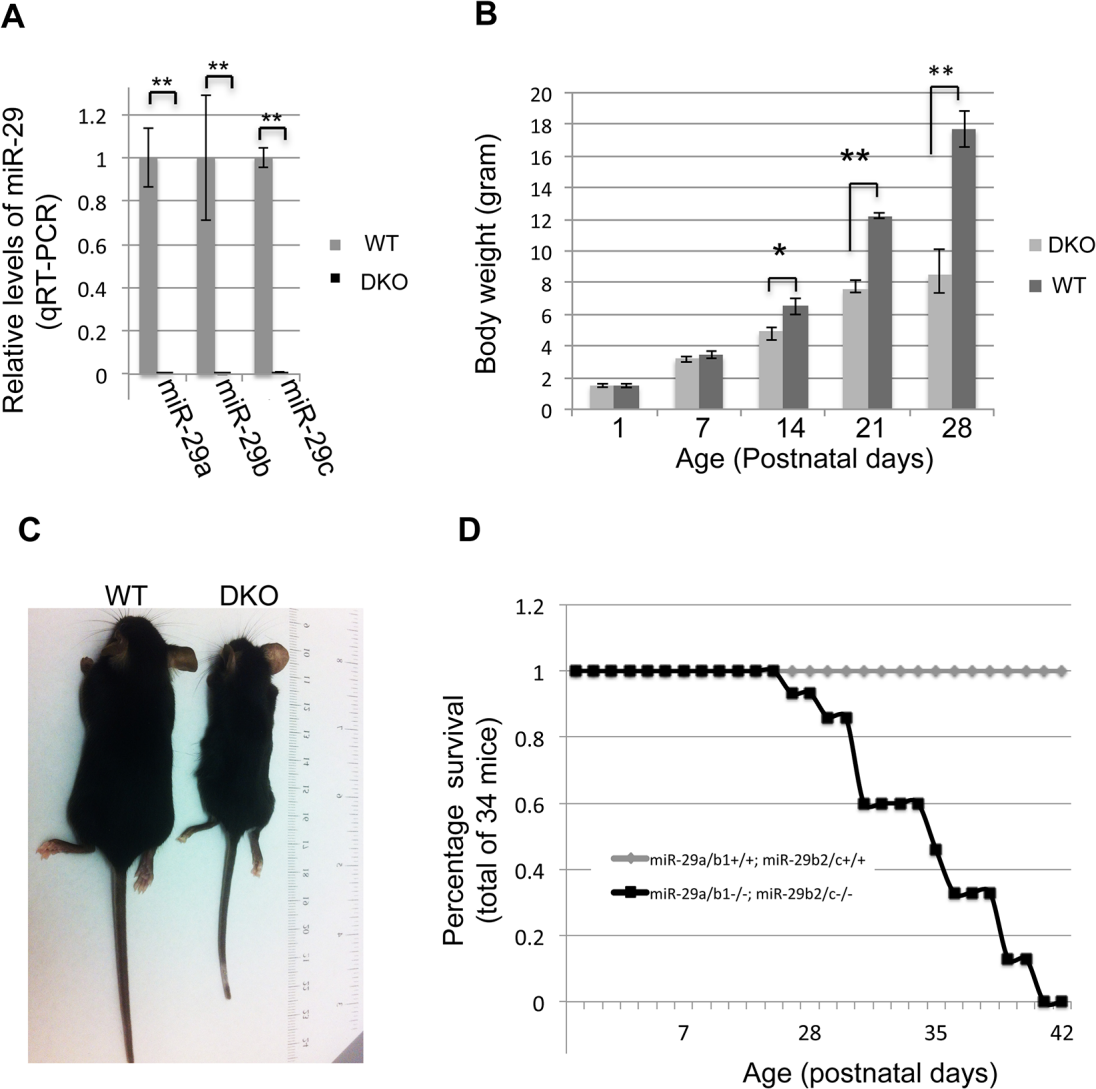
Postnatal growth retardation and lethality of miR-29 null mice. **(A)** Expression of miR-29a/b/c is undetectable in lungs of miR-29 DKO mice (n = 3). **(B)** miR-29 null mice are significantly smaller as compared to their littermates two weeks after birth (n = 5). **(C)** Representative image of miR-29 null mice at age four weeks. **(D)** Survival cure of miR-29 null mice (n = 15 for DKO and n = 19 for WT). All miR-29 null mice die within 6 weeks of birth, while none of wild type littermates died in the same period. Data are mean ± SEM; Student’s *t* test, * P<0.05; ** P<0.01.

Since expression of miR-29 family members (miR-29a/b/c) transcribed from both loci are enriched in vSMCs (Fig 1F), we decided to investigate the role of miR-29 *in vivo* by generating mutant mice in which both loci were deleted (double knockout or miR-29 null mice). Double knockout mice (DKO) were born with a normal Mendelian ratio and there is no obvious developmental abnormality at birth, as compared to their littermates. However, we observed a significant postnatal growth retardation, and miR-29 DKO are consistently smaller with about 25%, 40% and 50% reduction of body weight at ages of two, three and four weeks, respectively (Fig 3B and 3C). miR-29 null mice began to die around 4 weeks of age and none of them survived to the age of 6 weeks (Fig 3D).

**Fig 4:**
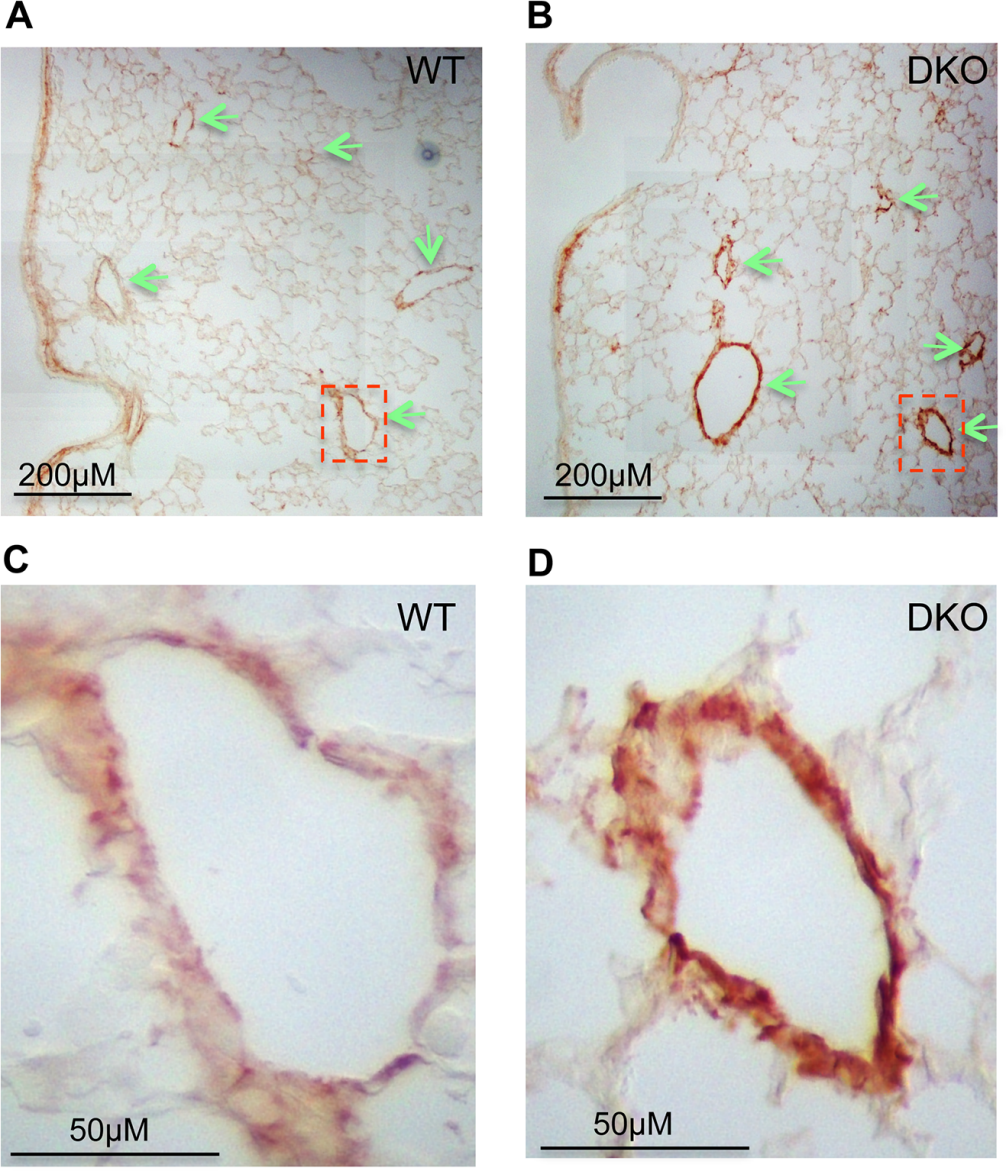
Upregulation of COL1A1 in distal vasculature of miR-29 DKO lungs. **(A&B)** Increased COL1A1 IHC signal is prominently associated with vessel walls, where endogenous miR-29 expression is highly expressed. **(C&D)** are enlarged images of highlighted areas of **A&B** by dashed line. All images are representative of four pairs of littermate-matched WT/DKO mice.

**Fig 5:**
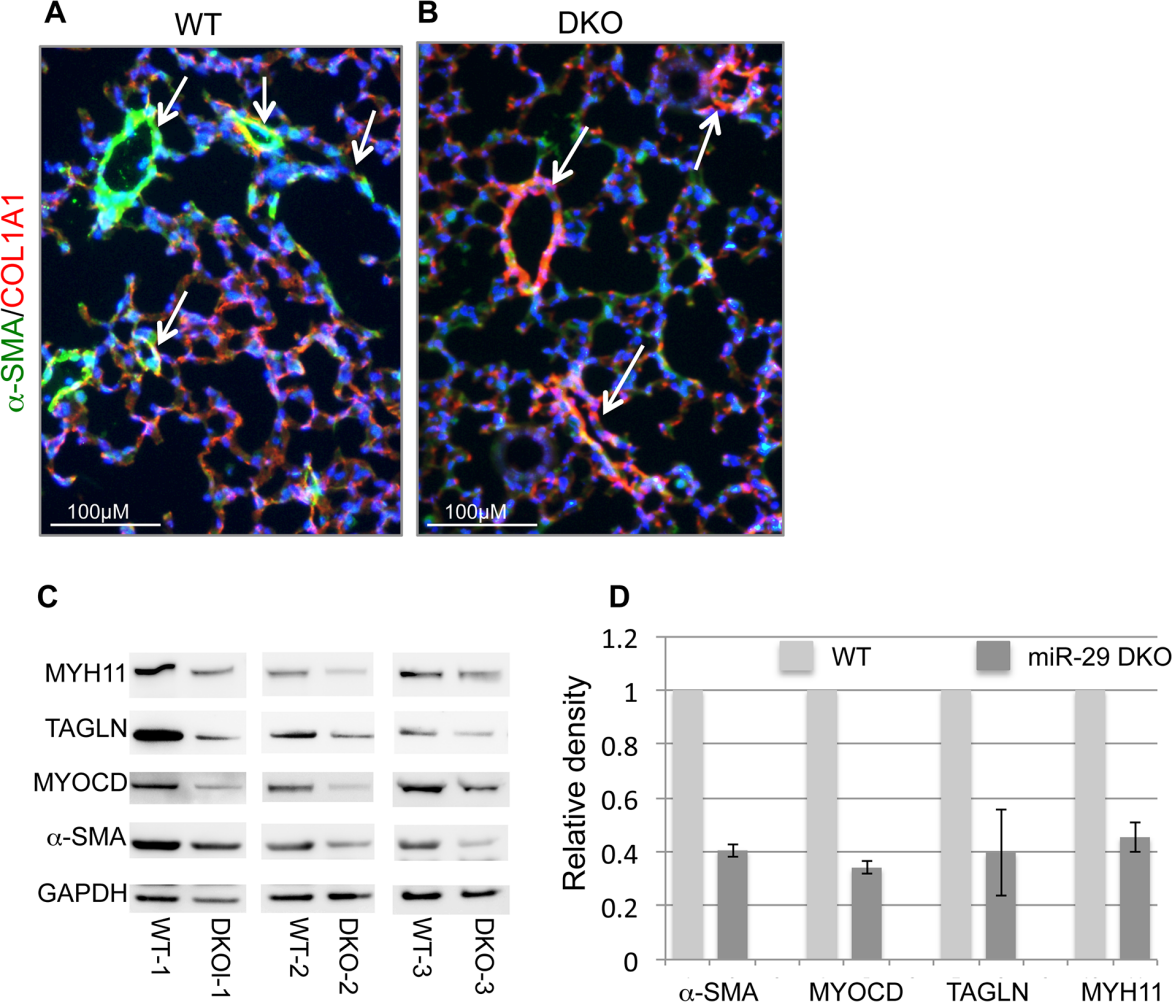
Disruption of miR-29 expression *in vivo* leads to aberrant vSMC differentiation. **(A&B)** Double IF staining of α-SMA and COL1A1; significantly reduced α-SMA staining (green) in distal vasculature was observed in miR-29 null lungs (DKO) as compared to wild type littermate controls (WT). This reduced expression of α-SMA is associated with upregulation of COL1A1(red), a known direct target of miR-29. Images are representative of four pairs of littermate-matched WT/DKO. **(C)** Immunoblotting analysis of α-SMA, MYOCD, TAGLN and MYH11 of whole lung protein extracts of three littermate-matched WT/DKO pairs. **(D)** Densitometric analyses of the blots are presented as relative ratios of specific protein/GAPDH. Ratio of the WT control is arbitrarily presented as 1. Data are mean ±SEM from 3 experiments.

Examination of the lungs of miR-29 DKO mice at age four weeks revealed significant defect in differentiation of vSMCs. First, we examined whether targets of miR-29 are derepressed in DKO lungs by staining COL1A1, a well-known target of miR-29 [22,23]. As expected, and consistent with miR-29 expression pattern, we found that the prominent upregulation of COL1A1 is associated with distal vessel walls of miR-29 null lungs (Fig 4A–4D). No significant upregulation of COL1A1 in the media layer of the aorta walls and in airway SMCs was detected (S1C and S1D Fig, S2A and S2B Fig). We then performed double immunofluorescence staining (IF) of α-SMA and COL1A1, and found that levels of α-SMA within vessel walls of distal vessels are significantly reduced, where COL1A1 staining is increased (Fig 5A and 5B). In contrast, levels of α-SMA in airway SMCs or within the media layer of the aorta are not significantly affected (S1E and S1F Fig, S2C and S2D Fig). We then performed immunoblotting analysis of the whole lung protein extract, and observed a significant reduction of levels of α-SMA, Myocardin (MYOCD), Transgelin (TAGLN) and Myosin, heavy chain 11 (MYH11), additional contractile markers of SMCs (Fig 5C and 5D). These findings suggest that miR-29 is specifically required for the proper differentiation of vSMCs of distal lung vasculature during development.

### Endogenous miR-29 is required for proper SMC differentiation *in vitro*

**Fig 6:**
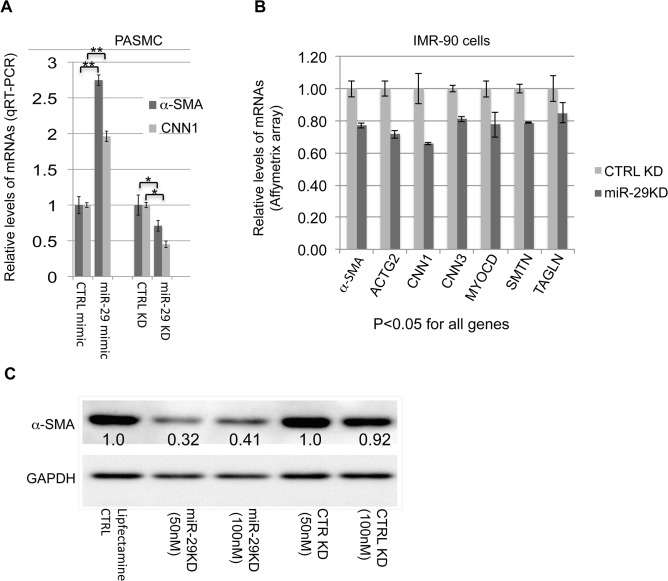
miR-29 promotes the expression of contractile SMC markers. **(A)** qRT-PCR results of α-SMA and CNN1 mRNAs in PASMCs in which the level of miR-29 is either elevated by miR-29 mimic or knocked down by miR-29 antisense LNA oligos (n = 3); **(B)** mRNA levels of contractile SMC markers in IMR-90 cells, in which miR-29 is knocked down (n = 3, Affymetrix array data); **(C)** Levels of α-SMA protein in IMR-90 cells, in which endogenous miR-29 is knocked down. Densitometric analyses of the blot are presented as relative ratios of α-SMA /GAPDH. Ratio of the control is arbitrarily presented as 1. Fold changes of ratio are indicated beneath the corresponding bands. Data are mean ± SEM; Student’s *t* test, * P<0.05; ** P<0.01.

Since both miR-29 loci were systemically deleted in DKO mice, the observed vSMC phenotype may results of secondary or accumulated causes. Due to the difficulty in isolation and culture of mouse lung SMCs, and also based on the selectively enriched expression of miR-29 in vSMCs of human lungs, we decided to investigate the role of miR-29 in human pulmonary arterial smooth muscle cells (PASMCs). We first manipulated miR-29 levels in PASMCs by transfecting with miR-29 mimic or LNA antisense oligos. We found that knocking down levels of endogenous miR-29 resulted in about 25–50% reduction of both α-SMA and calponin1 (CNN1), while increased miR-29 levels resulted in about a two fold upregulation of α-SMA and CNN1 in PASMCs (Fig 6A). This strongly suggests that endogenous miR-29 promotes the expression of vSMC contractile markers.

Previously, we have carried out Affymeritx array profiling for downstream genes of miR-29 in human fetal lung fibroblast cells (IMR-90) [23]. IMR-90 are myofibroblast cells that highly express many SMC markers including α-SMA and TAGLN[24]. We then examined the expression of SMC-related genes in this array data. This analysis revealed that knockdown of endogenous miR-29 results in 20–35% reduction of about 15 smooth muscle- and actin cytoskeleton-related genes including well-known SMC markers (all p<0.05), such as CNN1, CNN3 (calponin3), ACTG2 (actin, gamma 2, smooth muscle, enteric), ACTA2 (α-SMA), SMTN (smoothelin), TAGLN, as well as MYOCD, a master regulator of SMC gene expression (Fig 6B and S1 Table). In addition, Western Blot analysis showed a more than 60% reduction in α-SMA protein in miR-29 knockdown cells (Fig 6C).

### KLF4 is a direct target of miR-29 in SMCs

**Fig 7:**
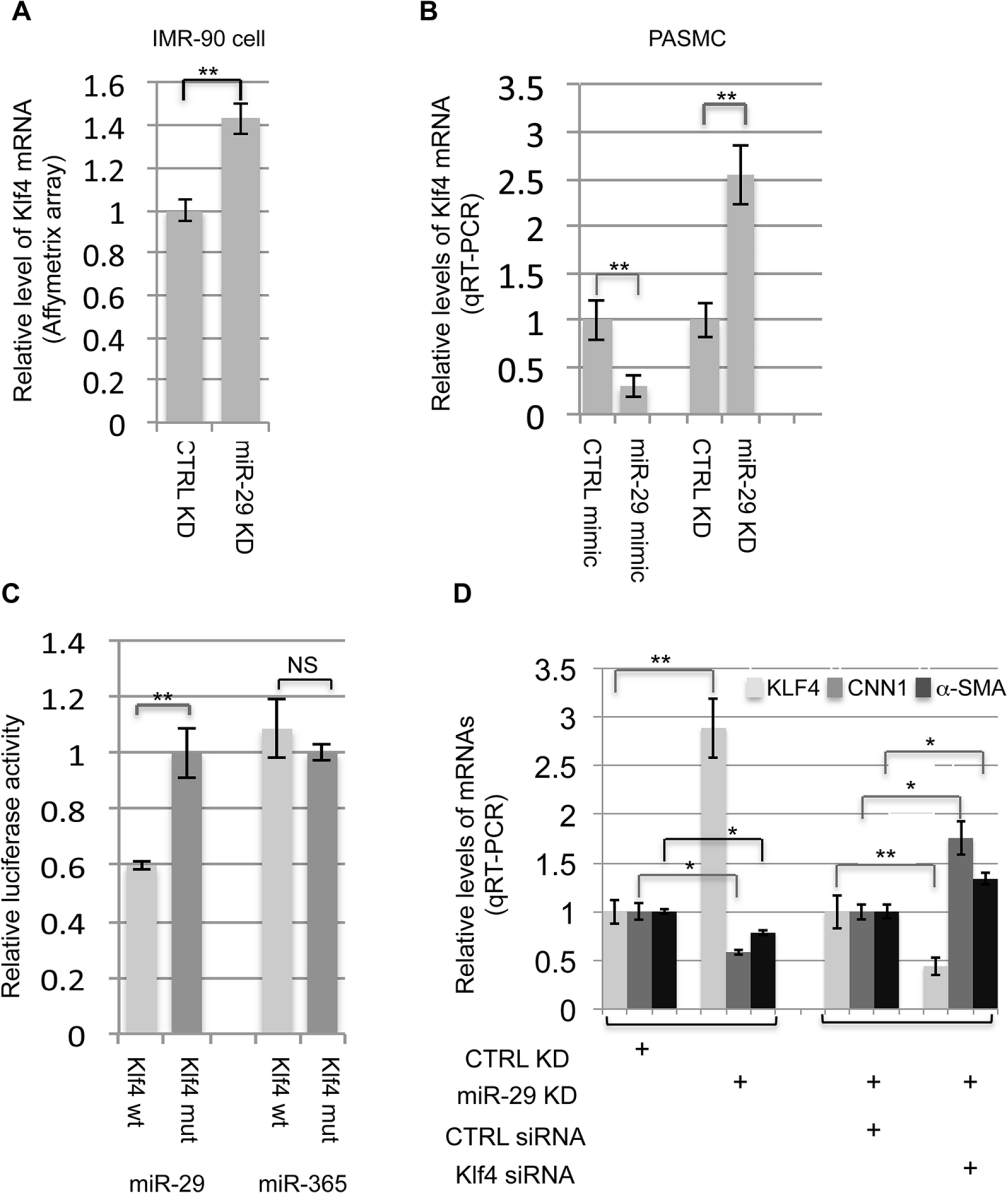
miR-29 regulates KLF4. **(A&B)** Knockdown of miR-29 in IMR-90 cells or PASMCs leads to upregulation of KLF4 expression (n = 3); increased miR-29 levels by mimic in PASMCs suppress the expression of KLF4 (n = 3); **(C)** miR-29 suppresses 3’UTR luciferase reporter of KLF4 which depends on an intact miR-29 binding site (n = 3); **(D)** Knockdown of KLF4 in PASMCs, in which miR-29 is also knocked down, restore the expression of CNN1 and α-SMA (n = 3). Data are mean ± SEM; Student’s *t* test, * P<0.05; ** P<0.01.

Our next inquiry was to determine how miR-29 up-regulates the expression of SMC-related genes. In general, miRNAs are negative regulators of their targets, therefore, we hypothesized that miR-29 indirectly upregulates the expression of SMC-related genes by targeting a negative regulator of SMC differentiation. We first turned to our array data of IMR-90 cells, in which endogenous miR-29 was knocked down. We found that the level of KLF4, a known negative regulator of SMC differentiation, is significantly increased (more than 40%, P<0.01) in miR-29 knockdown cells (Fig 7A). KLF4 negatively regulates the expression of smooth muscle related genes by interfering with the binding of SRF to their promoters[10,25]. We then investigated whether KLF4 is under the control of miR-29 in PASMCs, and found that over-expression of miR-29 with its mimic resulted in a two fold reduction of KLF4 mRNA, while knockdown of miR-29 resulted in about a two fold upregulation of KLF4 expression in PASMCs (Fig 7B). Together, results from these two cell lines suggest that expression of KLF4 is under the control of miR-29.

We then investigated whether miR-29 directly target KLF4 to exert its effects on SMC gene expression. Interestingly, KLF4 is a predicted target of miR-29 (TargetScan) and the binding site of miR-29 within 3’UTR of KLF4 is evolutionarily conserved among all species of vertebrates (total 22 species, TargetScan). To determine whether KLF4 is a direct target of miR-29, we conducted a 3’UTR luciferase assay. A fragment of the 3’UTR of KLF4 containing the wild type or mutated miR-29 binding sites was cloned into the psiCHECK2 dual luciferase reporter plasmid. Luciferase reporters were co-transfected with miR-29 mimic or miR-365 mimic, an unrelated miRNA as negative control. Luciferase reporter analysis revealed that the activity of reporter containing wild type 3’UTR of KLF4 was significantly suppressed (about 40% reduction) by miR-29, but not by miR-365 (Fig 7C). This suppression was abolished by mutating the miR-29 complementary binding site in KLF4 3’UTR(Fig 7C). This confirmed that KLF4 is a direct target of miR-29.

### Suppression of KLF4 rescues the defective SMC phenotype of miR-29 knockdown cells

We then examined whether reduction of KLF4 by siRNA can rescue defects in the expression of contractile markers of miR-29 knockdown cells. To do this, we repeated the experiment, in which miR-29 was knocked down alone in PASMCs. As expected, knockdown of miR-29 alone results in more than a two fold upregulation of KLF4 and significantly reduced expression of α-SMA and CNN1 (Fig 7D). Then, we examined whether reduction of KLF4 by siRNA can restore the expression of α-SMA and CNN1 in miR-29 knockdown PAMSCs. This analysis showed that upregulation of KLF4 by miR-29 knockdown is reversed by co-transfection of KLF4 siRNA, which leads to even lower KLF4 levels, as compared to cells of miR-29 knockdown alone. Furthermore, levels of α-SMA and CNN1 are no longer reduced when KLF4 is reduced by siRNA (Fig 7D). Instead, mRNA levels of both CNN1 and α-SMA in these cells are 30–70% higher than those of cells with miR-29 knockdown alone, negatively correlated with reduced KLF4 expression. This strongly suggests that derepression of KLF4 contributes to the negative regulation of SMC differentiation in miR-29 knockdown cells.

### Disruption of miR-29 leads to upregulation of Klf4 expression in vSMCs *in vivo*

**Fig 8:**
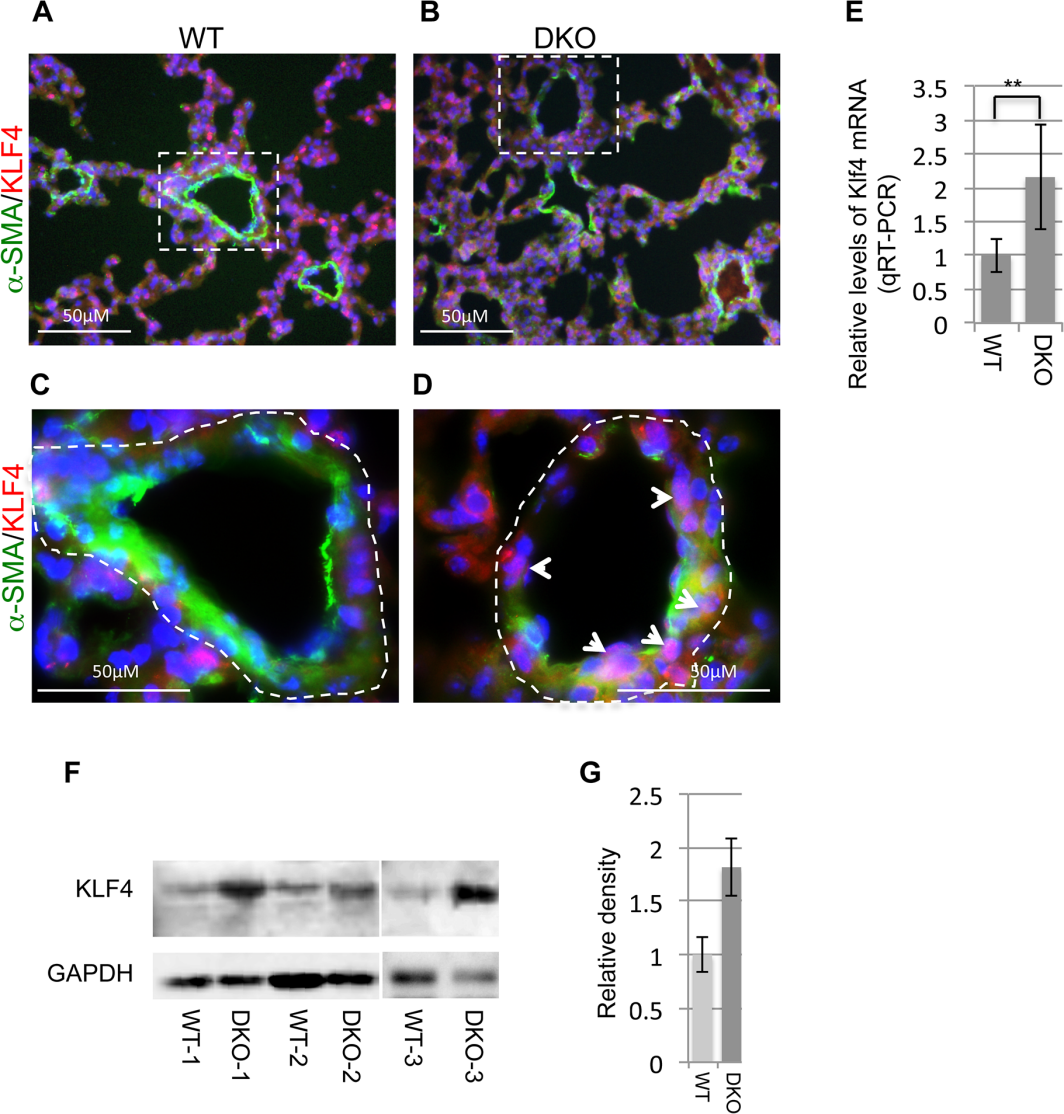
Upregulation of KLF4 in miR-29 null lungs. **(A&B)** Double IF staining of KLF4 and α-SMA; reduced vasculature α-SMA staining (green) is associated with increased KLF4 staining in the nucleus of cells associated with vessel walls (white arrowheads in **D**). **(C&D)** are enlarged images of highlighted areas of **A&B**. Images are representative of four littermate-matched WT/DKO pairs. **(E)** Levels of Klf4 mRNA in lungs of wild type and DKO mice (n = 3). **(F)** Western blot of KLF4 of whole lung protein extracts from three pairs of littermate-matched WT and DKO mice. **(G)** Densitometric analyses of the blot are presented as relative ratios of KLF4 /GAPDH. Ratio of WT lungs is arbitrarily presented as 1. Data are mean ± SEM; Student’s *t* test, ** P<0.01.

We then investigated the expression of Klf4 in miR-29 null mouse lungs. By double immunofluorescence staining of KLF4 and α-SMA, we found significantly increased Klf4 staining in the nucleus of cells associated with distal vessel walls of miR-29 null lungs, inversely correlated with the reduced staining of α-SMA (Fig 8A–8D). This upregulation of KLF4 expression is specific to distal muscular vessels, and levels of KLF4 in the media layer of aorta, and in airway smooth muscle cells remaining unchanged (S1E and S1F Fig, S2C and S2D Fig). By qRT-PCR, we detected a two fold increase in Klf4 mRNA in miR-29 null lungs, as compared to those of littermate controls (Fig 8E). This was further confirmed by western blot analysis (Fig 8F and 8G), in which protein levels of KLF4 of miR-29 null lungs are significantly higher than those of control littermates. Together, these results for the first time showed that KLF4 is a physiological target of miR-29 *in vivo*, and increased KLF4 in distal vSMCs of miR-29 null lungs likely contribute to the immature vSMC phenotype.

### Disruption of miR-29 leads to upregulation of components of the PDGF pathway

**Fig 9:**
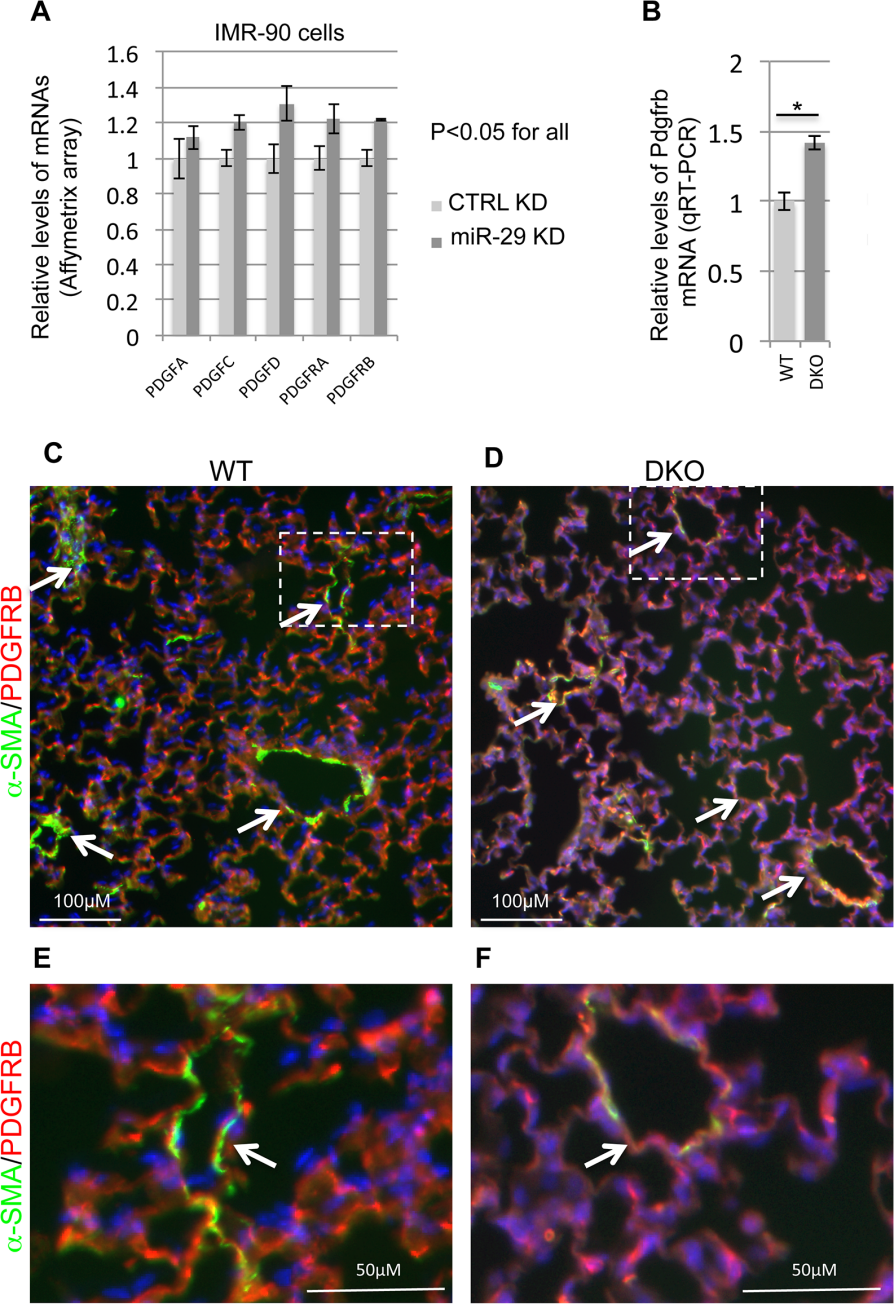
Upregulation of components of PDGF pathway in both miR-29 knockdown cells and miR-29 null lungs. **(A)** Affymetrix array data of components of PDGF signaling pathway in miR-29 knockdown cells (n = 3). **(B)** qRT-PCR of Pdgfrb mRNA in lungs of wild type and DKO mice (n = 3). **(C&D)** Double IF staining of PDGFRB (red) and α-SMA (green) in WT control and miR-29 DKO lungs. **(E&F)** are enlarged images of areas highlighted by white dashed lines of **C** and **D**. Reduced vasculature α-SMA staining (green) is associated with increased PDGFRB staining (white arrowheads) in cells associated with vessel walls in DKO lungs (**D&F**). Images are representative of four littermate-matched WT/DKO pairs. Data are mean ± SEM; Student’s *t* test, * P<0.05.

Another major negative regulator of SMC differentiation is PDGFB[26,27]. In cultured SMCs *in vitro*, PDGFB can induce a profound phenotype switch by suppressing the expression of SMC specific genes at both the transcriptional and posttranscriptional levels mainly via PDGFRB[26,28]. Interestingly, results from different cell types showed a potential reciprocal negative feedback loop of miR-29 and components of the PGDF pathway. While expression of miR-29 is suppressed by treatment of PDGFB, miR-29 is known to directly target the expression of components of the PDGF pathway, such as PDGFRB[ 29 – 31 ]. Most recently, it has been shown in a vascular injury model that upregulation of miR-29 suppresses the expression of PDGFRB in modulating SMC phenotype [ 32 ]. To ask whether the PDGF pathway is under the control of miR-29 in vSMCs, we first examined their expression in our array data of miR-29 knockdown IMR-90 cells. Interestingly, knockdown of miR-29 leads to significant upregulation of all PDGF ligands and receptors (15%-30% upregulation, p<0.05), except for PDGFB (Fig 9A). This likely leads to increased PDGF signaling activity. We then performed qRT-PCR to examine the levels of Pdgfrb in miR-29 null or wild type lungs. We found that the level of Pdgfrb is significantly upregulated in miR-29 null lungs (Fig 9B). To examine the levels of PDGFRB in distal vessel wall of miR-29 null lungs, we performed co-immunofluorescence staining of α-SMA and PDGFRB. This analysis revealed much higher PDGFRB staining in distal vessel walls of miR-29 null lungs, negatively correlated with reduced signal of α-SMA staining (Fig 9C–9F). Together, our results suggested that miR-29 suppresses the PDGF pathway in promoting vSMC differentiation during lung development *in vivo*.

### Levels of Foxo3a and Fbxo32 are significantly upregulated in miR-29 deficient cells

**Fig 10:**
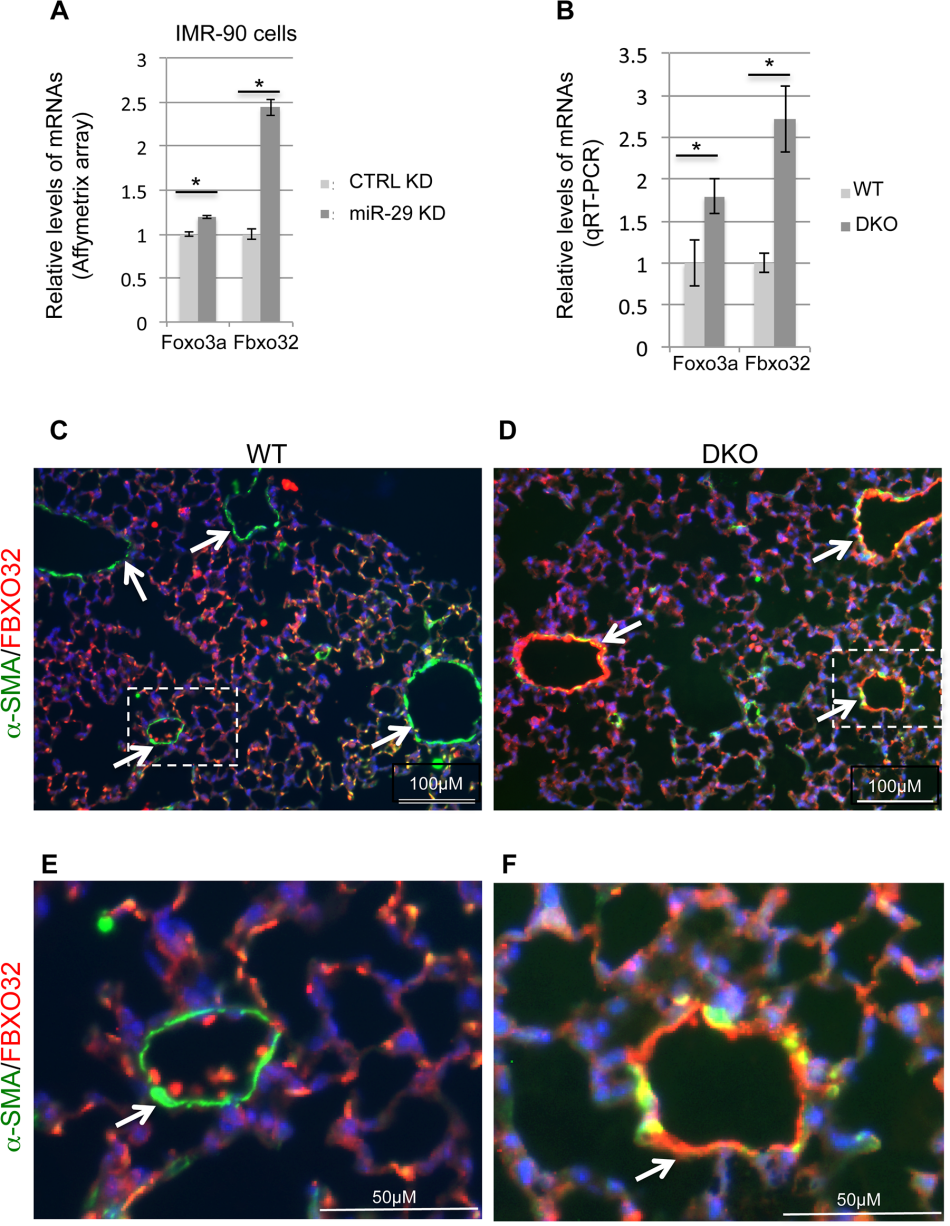
Upregulation of Foxo3a and Fbxo32 in both miR-29 knockdown cells and miR-29 null lungs. **(A)** Affymetrix array data of Foxo3a and Fbxo32 in miR-29 knockdown cells (n = 3). **(B)** qRT-PCR of Foxo3a and Fbxo32 mRNAs in lungs of wild type and DKO mice (n = 3). **(C&D)** Double IF staining of FBXO32 (red) and α-SMA (green) in WT control and miR-29 DKO lungs. **(E&F)** are enlarged images of areas highlighted by white dashed lines of **C** and **D**. Reduced vasculature α-SMA staining (green) is associated with increased FBXO32 staining (white arrowheads) in cells associated with vessel walls in DKO lungs (**D&F**). Images are representative of four littermate-matched WT/DKO pairs. Data are mean ± SEM; Student’s *t* test, * P<0.05.

One of the most upregulated genes in miR-29 knockdown human lung myofibroblast cells is FBXO32 (Atrogin-1), together with its activator FOXO3a (Fig 10A). FBXO32 is a muscle-specific ubiquitin ligase, well known for its role in muscle wasting of skeletal muscle and in the inhibition of hypertrophy of cardiac myocytes[33]. Expression of FBXO32 is enriched in skeletal, cardiac, and smooth muscle[34]. FOXO3a/FBXO32 has been shown to be involved in the direct degradation of the BK (Big Potassium) channel β1 subunit in diabetic mice or diabetic human vascular smooth muscle cells, a key subunit of Ca2+-activated K+ (BK) channels which is important for proper vascular function[35–37]. FBXO32 is expressed in uterine smooth muscle cells and its level is drastically upregulated in association with involution. FOXO3a is known to play a role in the vascular smooth muscle cell proliferation and apoptosis down stream of the PI3K-AKT pathway[38–40]. Together, this suggests a role for FOXO3a/FBXO32 in smooth muscle cells. We first performed qRT-PCR for Foxo3a and Fbxo32, and found that both of them are significantly upregulated in miR-29 null lungs, as compared to littermate controls (Fig 10B). We then performed co-immunofluorescence staining of α-SMA with FBXO32, and found that levels of FBXO32 are drastically upregulated specifically in the distal vessel walls of miR-29 null lungs, negatively correlated with the reduced α-SMA staining (Fig 10C–10F). This upregulation of FBXO32 is not presented in SMCs of airway and large proximal vessels, consistent with the expression pattern of miR-29 (S4A and S4B Fig). Since there is no predicted miR-29 binding site in the 3’UTR of FBXO32 mRNA, it is likely that miR-29 indirectly suppresses the expression of FBXO32 by targeting Foxo3a, which is also upregulated in miR-29 knockdown cells. A evolutionarily conserved miR-29 binding site is present in the 3’UTR of Foxo3a (Targetscan). While we were working on this project, it was reported that Foxo3a is a direct target of miR-29 during chondrogenic differentiation[41]. Together, our results identified that FBXO32 is a novel downstream gene of miR-29, suggesting miR-29 might regulate smooth muscle protein degradation by modulating the expression of Foxo3a/Fbxo32.

## Discussion

miR-29 is known for its roles in fibrosis, cell proliferation/apoptosis, tumor and adaptive and innate immunity [42–54]. However, little is known regarding the expression and function of miR-29 during development *in vivo*. Here, we report that miR-29 is required for postnatal growth and development. In distal lung vSMCs, miR-29 promotes the differentiation of vSMCs by targeting Klf4 and the PDGF pathway, two major negative regulators of vSMC maturation. Moreover, our work also suggests a novel role of miR-29 in regulating the expression of Foxo3a/Fbxo32 in distal vSMCs.

The postnatal growth retardation and lethality are coincident with the drastic upregulation of miR-29 expression in multiple organs during postnatal development. These include aorta [55], skeletal muscle[56], epididymis[57], lung[23], brain[58] and cornea[59]. However, the exact cause of this phenotype is unclear. Due to the nature of systemic knockout of miR-29 in animals examined in this study, and diverse functions of miR-29 in different cell types and organs, failure of multi-organs might contribute to the growth retardation and lethality. Most of these miR-29 null mice develop a hunchback, indicating weakness of the skeletal muscles. It has been showed that down-regulation of miR-29 is associated with dystrophic muscles[54,60] and restoration of miR-29 expression improved dystrophy pathology[60]. This might be due to the upregulation of Foxo3/Fbxo32 in the skeletal muscle of miR-29 null mice, which requires careful examination. Another possibility is cardiac failure due to the abnormal distal lung vasculature. However, by examining the weight of hearts of miR-29 null and wild type littermates, there is no significant sign of heart failure. In fact, hearts of miR-29 null mice are about 34% smaller as compared to those of control, by normalizing to tibial length (S5A and S5B Fig).

Expression of miR-29 in mouse lungs is abundant, selective and dynamic. Previously we showed that levels of miR-29 are significantly upregulated during embryonic and postnatal lung development that was confirmed by Next Generation of Sequencing and qRT-PCR [23] (S2 Table and S3 Fig). In this study, we found that miR-29 expression is selectively enriched in vSMCs of distal vessels of mouse lungs, a pattern that is also conserved in human lungs. SMC-specific miRNAs have been identified, such as miR-143/145. The level of miR-145 is also abundant (S2 Table) in adult mouse lungs. miR-143/145 are expressed in both airway SMCs and vSMCs [61]. Unlike miR-29, there is little change in levels of miR-143/145 during embryonic and postnatal development (S2 Table). The most drastic upregulation of miR-29 (S2 Table and S3 Fig, about 20–40 folds) happens during postnatal development, a period in which distal vSMCs gradually switch from synthetic to contractile phenotypes in human or animal lungs. This indicates a critical role of miR-29 in the postnatal vSMC maturation.

miRNAs are known to play critical roles in vSMC differentiation and contractile function. Deletion of *Dicer1* by *SM22α-Cre* or *SMMHC-CreERT2 in vivo* resulted in poorly differentiated vSMCs, leading to abnormal vessel wall and extensive hemorrhage [18,19]. These findings were supported by similar vSMC defects in animals, in which *Drosha* or *DGCR8*, two key components of pri-miRNA processing, are specifically deleted [62,63]. Together, these findings suggest critical roles of miRNAs for the differentiation of SMCs. miR-143/145 has been shown to play an important role in SMC development *in vivo* [20,61,64]. However, the vSMC phenotype of miR-143/145 null mice is much milder as compared to those of *Dicer1* mutant[19,20]. Major vSMCs defects of miR-143/145 null mice are disarray of actin stress fibers and diminished migratory activity of SMCs. There is no significant defect in the expression of contractile SMC markers in miR-143/145 null mice[20,61]. Thus suggesting additional miRNAs must be required for proper differentiation of vSMCs *in vivo*. In this study, we found that the expression of contractile SMC markers is significantly attenuated in miR-29 null lungs, preferentially affecting vSMCs of distal pulmonary vasculature. In addition, we also found potent activity of miR-29 in promoting SMC differentiation *in vitro*. Together, these results suggested that miR-29 is part of the miRNA regulatory network in promoting SMC differentiation.

Our data suggested that miR-29 promotes SMC differentiation at least partially by suppressing KLF4 and components of the PDGF signaling pathway. In the course of our study, KLF4 proved to be the direct target of miR-29 in cancer cell lines [65,66]. Here we showed that KLF4 is directly targeted by miR-29 in vSMCs and our data indicates that Klf4 is a physiological target of miR-29 *in vivo*. KLF4 is a potent negative regulator of SMC differentiation by interfering with the activity of SRF [10,25]. KLF4 also suppresses the expression of MYOCD, an important transcriptional factor in promoting SMC differentiation [25,67]. This is consistent with the decreased expression of MYOCD and a large number of SMC contractile markers in miR-29 knockdown cells and miR-29 null lungs (Fig 5 and Fig 6). miR-29 also plays a critical role in regulating the PDGF pathway in multiple cell types [29,32]. Moreover, it has been shown *in vitro* that MYOCD induces the expression of miR-24 and miR-29 and promotes SMC differentiation by suppressing PDGFRB [32]. Indeed, in miR-29 knockdown IMR-90 cells, expression of all components of the PDGF pathway, except for PDGFB, is significantly upregulated (Fig 9A). In this study, we also showed that Pdgfrb is a physiological target of miR-29 *in vivo* (Fig 9B–9F). miR-29 also directly suppresses the expression of ECM-related genes associated with synthetic phenotypes, such as collagen [22,23,68]. Together, our study showed that miR-29 is a central player in promoting SMC differentiation by suppressing the expression of KLF4, PDGFRB and ECM-related synthetic genes.

Both SMC-specific deletion of *Dicer1* and systemic miR-143/145 knockout compromise vascular contractile function and result in lower blood pressure[18,20,64,69]. Moreover, miR-143/145 null mice are protected from hypoxia induced pulmonary hypertension[69]. The early postnatal lethality and the small size of miR-29 DKO mice, prevent us from measuring blood pressure. We found that there is a significant reduction in heart weight, left ventricle weight and right ventricle weight when normalized to tibial length in miR-29 null mice (S5B Fig). This indicates a reduction of heat size in miR-29 null mice. However, there is no significant difference between miR-29 null and control mice in heart weight when normalized to body weight (S5C Fig). Together, these analyses suggest that defects in vSMC differentiation in miR-29 null lungs, might lead to the reduction in pulmonary arterial pressure. Distal pulmonary vSMCs are known to behave differently in response to hypoxia, one of the important environmental factors and common causes of pulmonary arterial hypertension. Moreover, it is known that populations of vSMCs adopt a dedifferentiated or immature phenotype with increased activities of proliferation, migration and ECM-production in the early stage of development of pulmonary arterial hypertension (PAH) [3,5,70–72]. However, PAH is characterized in general by the extension of vSMCs to distal arteries, increased vSMC mass and contractility. The immature/synthetic vSMC phenotype of distal lung vessels of miR-29 null mice indicates a potential dysregulation of miR-29 in the pathogenesis of PAH. Moreover, by targeting a large number of ECM-related genes, miR-29 plays a significant role in collagen vascular diseases that has been increasingly recognized as potential causes for the development of PAH. Together, these results suggest that it is highly relevant to examine the expression and function of miR-29 in vSMCs of pulmonary vascular diseases.

## Materials and Methods

### Generation of miR-29 knockout mice

Targeting strategies and procedures of generation of both floxed *miR-29ab1* and floxed *miR-29b2c* mice have been described in detail by Smith KM et. al. [53]. Germ line deletions for both alleles were achieved by breeding *miR-29ab1* ^flox/flox^ mice or *miR-29b2c* ^flox/flox^ mice to EIIa-cre mice. This allows establishing systemic knockout mice for *miR-29ab1* and *miR-29b2c* alleles. *miR-29ab1* ^+/-^; *miR-29b2c* ^+/-^ mice are viable and fertile, and were used to breed for generation of double knockout (miR-29 null) mice with mixed genetic background containing 129/SvJ and C57BL/6. The health condition of litters were monitored daily, and their body weight was measured once a week in the first 5 weeks. Sequences of primers used for genotyping and size of PCR products are: miR-29ab1-common-forward, 5’-TGTGTTGCTTTGCCTTTGAG-3’, with miR-29ab1-WT-reverse, 5’-CCACCAAGAACACTGATTTCAA-3’ (wild-type band, 450bp), or miR-29ab1-KO-reverse, 5’CGAATTCGCCAATGACAAGACGCT-3’ (KO band, 550bp); miR-29b2c-common-forward, 5’-TGCTAGCCTACAGGGTCATGG-3’, with miR-29b2c-WT-reverse, 5’-TGTCAGACGCAGATGACAGC-3’ (wild-type band, 350bp), or miR-29b2c-KO-reverse, 5’CGAATTCGCCAATGACAAGACGCT-3’ (KO band, 550bp).

### Lung cell dissociation, cell sorting and flow cytometry

Cell dissociation was achieved by digesting mouse lungs from transgenic mice of α-SMA-EGFP or T1a-EGFP with a commercially available Lung Digestion Kit (Miltenyi Biotech, Auburn, CA). Cells were further stained with anti-mouse CD34 (BD Biosciences) or CD31 antibodies (BD Pharmingen, San Diego, CA). Isotype antibodies were used as controls. SMCs (α-SMA-EGFP), or type I epithelial cells (T1a-EGFP) or hematopoietic cells (CD34 positive) or endothelial cells (CD31 positive) were collected using a MoFlo cell sorter (Beckman Coulter, Fullerton, CA).

### Reagents and cell culture

Human fetal lung fibroblasts (IMR-90) and human pulmonary arterial smooth muscle cells (PASMCs) were cultured and transfected with miR-29 mimics, miR-29 LNA antisense or KLF4 siRNA as described [23,73]. Cell culture was performed in a biological safety hood with sterile techniques employed. IMR-90s (cat. #ATCC CCL-186) were cultured in DMEM media (Life Technologies) supplemented with 10% FBS, 100 units penicillin/100 mL and 100 μg streptomycin/100 mL. PASMCs (Lonza, CC-2581) were cultured in SMGM-2 media (Lonza, CC-3181 & CC-4149) supplemented with 5% FBS, 0.01% insulin, 0.02% gentamicin/amphotericin-B, 0.02% hFGF-B and 0.01% hEGF. All cells were maintained in a humidified 5% CO2, 95% air incubator at 37°C. Cells were passaged and split by first washing with PBS and then using trypsin/EDTA (Life Technologies) to dislodge cells. Cells were then resuspended and plated in fresh media. Lipofectamine 2000 (Life Technologies, Cat.# 11668019) was used for all transfections according to the manufacturer’s protocol. We selected hsa-miR-29c-3p miRCURY LNA microRNA inhibitor (product#: 4105460–001, and sequence: CCGATTTCAAATGGTGCT) and miRCURY LNA microRNA inhibitor negative control (product#: 199007–001, sequence: AGAGCTCCCTTCAATCCAA) for knockdown assay based on our published data that hsa-miR-29c LNA antisense (50nM) can efficiently reduce the endogenous levels of all three miR-29 family members. hsa-miR-29c-3p mimic (Dharmacon, cat.# C300650-07) and microRNA Mimic Negative Control (Dharmacon, cat.# CN-001000-01) were transfected at 5nM for upregulation of miR-29 in cultured cells. ON-TARGET plus SMARTpool human KLF4 siRNA (Thermo Scientific, Cat.#9314) and negative control (ON-TARGET plus Non-targeting Pool, Cat.# D-001810-10) were transfected at a concentration of 100nM for the knockdown of KLF4.

### RNA isolation from cell culture and mouse lungs

Total RNA samples were isolated using TRIzol (Life Technologies, Cat.# 15596). Briefly, cells were lysed directly in 6-well culture plates, by applying 1 mL of TRIzol to each well, and then transferred to Eppendorf tubes. Chloroform was applied for phase separation and the mixture was vortexed and centrifuged at 12,000 X g for 15 minutes at 4°C. The upper aqueous phase containing the RNA was transferred to fresh Eppendorf tubes. The RNA was precipitated with isopropyl alcohol, the mixture was incubated at room temperature (RT) for 10 minutes and centrifuged at 12,000 X g for 10 minutes at 4°C. Supernatant was then removed and the RNA pellet washed with 75% ethanol by vortexing and centrifuging at 12,000 X g for 5 minutes at 4°C. Supernatant was again removed and the pellet briefly dried. RNA was dissolved in DEPC water and concentration measured by NanoDrop. For dissected mouse lungs, 1mL TRIzol were used for homogenizing 50mg of lung tissue. This was followed by similar procedures described above.

### Next-generation sequencing of small RNAs and data analysis

SOLiD system (ABI) was selected for sequencing, because of its high capacity for small RNAs and the data was analyzed by using CLC Genomics Workbench (CLC Bio). The sequencing was carried out in the Molecular Genetics Core Facility of Children's Hospital (Harvard University, Boston). Lungs from different developmental stages or adult mice were dissected for total RNA isolation. For each developmental stage, total RNA samples from two independent embryonic or adult lungs were processed separately for small RNA sequencing. First, small RNAs (less than 40nt) from 10μg of total RNA samples were isolated using FlashPAGE Pre-Cast Gels (life technologies, Type A, Cat #10010) and FlashPAGE Fractionator (Life Technologies). A range of 150ng to 300ng of small RNAs was isolated from total RNA samples. We then followed the standardized protocol of Small RNA Library Preparation for SOLiD Sequencing (ABI) to generate cDNA libraries. Briefly, small RNA samples were hybridized with Adaptor Mix with unique barcodes in reverse PCR primer for each cDNA library (SOLiD RNA Barcoding Kit, Module 1–16). This was followed by ligation of adaptors to the small RNA molecules using Ligation Enzyme Mix (ABI, SOLiD Small RNA Expression Kit). Next, the small RNA population with ligated adaptors was reverse transcribed to generate cDNA, followed by RNase H digestion to remove RNA from RNA/cDNA duplex. Then each cDNA library was amplified by PCR (16 cycles) using SOLiD 5’ PCR primer and a 3’ library-specific barcoded SOLiD primer. PCR amplified cDNAs were separated in 6% acrylamide gel stained with SYBR Gold (Invitrogen, Life Technologies) and PCR products with sizes of 105–150 bp were isolated, corresponding to sizes of small RNA inserts together with adaptor and primer. The resulting cDNA libraries were clonally amplified onto beads by emulsion PCR using the SOLiD ePCR Kit. Following the standard protocol provided by the manufacturer, libraries were deposited onto separated segments of a single slide and sequenced on the SOLiD Analyzer. Raw sequencing reads were analyzed by using the Genomic Workbench software platform (CLC Biosystems). First, sequences were sorted and grouped by specific barcodes for each cDNA library. The analysis generates about 10 to 12 million raw reads for each cDNA library. Then perfectly matched adaptor sequences were trimmed, while sequences without or with ambiguous adaptor sequences were discarded. After filtering for rRNA, tRNA, repeated sequences of mouse genome, the remaining reads were aligned to miRbase (Release 18) for known miRNAs. Reads with no more than two internal mismatches or no more than 3 mismatches at 3’ were selected and counted for known miRNAs. Reads per million were calculated for individual miRNAs for each library which allowed us to compare their expression levels.

### Quantitative qRT-PCR

TaqMan MicroRNA Assays (Life Technologies) for mmu-miR-29a (Cat.# 002112), mmu-miR-29b (Cat.#002497) and mmu-miR-29c (Cat.#001818) were used for qRT-PCR and U6 snRNA (Cat.# 4427975) was selected for internal control and normalization. 10 ng of total RNA per sample was subjected to reverse transcription using the TaqMan MicroRNA Reverse Transcription Kit (Life Technologies Cat. #4366596) and miRNA specific RT primers. TaqMan Universal PCR Master Mix and TaqMan microRNA Assays were used according to the manufacturer’s instructions. The qRT-PCR was performed on a StepOne Real-Time PCR System (Life Technologies). Delta-delta Ct method was used to determine the level of each miRNA relative to the level of endogenous U6 snRNA. For mRNAs, 1 μg total RNA per sample was subjected to reverse transcription using the SuperScript III First-Strand Synthesis System (Life Technologies, Cat.#18080) and oligo (dT) primer according to the manufacturer’s instructions. TaqMan Gene Expression Assays (Life Technologies) for human KLF4 (Cat.#Hs00358836_m1), mouse KLF4 (Cat.#Mm00516104_m1), human ACTA2 (Cat.#Hs00426835_g1), human CNN1 (Hs00154543_m1) were used for detection of mRNAs. Ribosomal protein L23 (RPL23, Cat. # 4351372) was used for normalization. The relative level of specific mRNA to RPL23 control was calculated by following the delta delta CT method.

### 3’ UTR luciferase assay

DNA fragments of the 3’UTR of Klf4 that host the predicted complementary binding site for miR-29 was amplified using genomic DNA from CD57BL/6 mice as a template by PCR (forward primer 5’ GACCAGAATTCCCTTGAATT 3’ and backward primer 5’ AAGTCAGGTAATATACCTGG). These were then cloned downstream of the Renilla luciferase reporter gene in the psiCHECK2 dual luciferase reporter plasmid (Promega, Cat.# C8021). Nested primers (forward: 5’CTAAATGATAATAATTGGTGAGTCTTGGTTCTA 3’ and reverse: 5’GACTCACCAATTATTATCATTTAGGCTATTTAAA 3’) were then used to mutate the binding site of miR-29 seed sequence by primer extension PCR. Human embryonic kidney (HEK) 293 cells were seeded in 96 well plates with DMEM (Life Technologies, Cat.#11995) and co-transfected with 10ng of luciferase reporter plasmid together with either 5nM of hsa-miR-29c-3p mimic (Dharmacon, Cat.#:C300650-07) or hsa-miR-365a-3p mimic (Dharmacon, Cat.#: C300666-03) using Lipofectamine 2000 (Life Technologies, Cat.# 11668019). miR-365a was selected as negative control, since there is no binding site of miR-365a in the cloned DNA fragment. Cells were lysed and luciferase activities were measured 48hrs after transfection, according to the manufacturer’s instructions using the Dual-Glo Luciferase Assay System (Promega, Cat. #E2920) and GloMax Multi-Detection System (Promega). Three independent experiments were performed and data was normalized to firefly luciferase activity expressed from the same psiCHECK-2 vector as an internal control.

### Protein preparation and western blot

Lane marker reducing sample buffer 5X (Life Technologies, Cat.#39000) was diluted to 1.0X with RIPA buffer. Cells were washed 3 times in cold PBS and 200 μL 1X SDS sample buffer per well (6-well plate) applied to cells. A cell lifter (Corning Costar) was used to mix and lyse the cells, which were subsequently transferred to Eppendorf tubes. Sonicated for 10–15 seconds and then heated for five minutes at 95°C, cooled on ice for 2 minutes and finally centrifuged at 8,000 X g for 1 minute. For dissected lung tissues, 100mg of tissue was homogenized in 1mL of 1X RIPA buffer (cell Signaling Technologies, Cat.#9806 with 1mM of PMSF freshly added before the experiment). Protein concentration was measured using BCA Protein Assay Kit (Cell Signaling Technology, Cat.#7780). 20–60 μg of Proteins were resolved on Tris-Glycine SDS 10% polyacrylamide gels, transferred to PVDF membranes using the iBlot dry blotting system (Invitrogen), and blocked with 5% nonfat dairy milk in Tris-buffered saline (20mM Tris, 150 mM NaCl, pH 7.4) with 0.1% Tween-20. Primary antibodies were diluted in 5% non-fat milk/1X PBST, and membranes were incubated with an antibody overnight at 4°C. Membranes were then washed 3 times for 10 minutes each in TBST. Horseradish peroxidase (HRP) conjugated secondary antibodies (Life Technologies) were diluted in 5% non-fat milk/1X PBST (1:2000) and incubated for 1 hour at room temperature. Membranes were again washed three times in TBST and blots were developed using Pierce ECL Western Blotting Substrate (Life Technologies, Cat.# 32209) or SuperSignal West Femto Maximum Sensitivity Substrate (Life Technologies, Cat.# 34095AB). Chemiluminescence signals were detected using LAS 4000 (GE Health Care) and ImageQuant (GE Health Care) were used for quantification of signal intensity. Band intensities were normalized against corresponding GAPDH and compared to wild-type controls for relative level. Primary antibodies for proteins including α-SMA (Sigma, Cat.#A2547, 1:2000), MYH11(Abcam, Cat.#ab53219, 1:500), TAGLN (Abcam, Cat.#ab10135, 1:500), KLF4 (Cell Signaling Technology, Cat.#4038, 1:50), MYOCD (R & D Systems, Cat.#MAB4028, 1:500) and GAPDH (R & D Systems, Cat.#AF5718, 1:2000) were used.

### Mouse lung dissection and embedding

To harvest adult lungs for analysis, perfusion was carried out to clear blood from the lungs by right atrium injection of 1 mL of PBS. Right lungs were cut for RNA or protein isolation, while left lung was intratracheally inflated with 4% paraformaldehyde, diluted from 16% of paraformaldehyde (VWR, Cat.#100503) at a constant pressure of 30 cm H_2_O, and used for subsequent frozen and paraffin embedding and sectioning. For frozen sections, tissues were fixed in 4% paraformaldahyde overnight at 4°C and then washed in 1X PBS for 30 minutes at 4°C, followed by 10% sucrose in 1X PBS for 1 hour at 4°C, then 15% sucrose in 1X PBS for 1 hour at 4°C, then 20% sucrose in 1X PBS for 1 hour at 4°C, finally followed by rotation in a 1:1 ratio of 20% sucrose:OCT overnight at 4°C. Tissues were then embedded in a 1:3 ratio of 20% sucrose:OCT on dry ice and sectioned in 8–12 μM sections using Cryostat (Leica), followed by mounting on glass slides, air drying at RT 1–2 hours and sections were stored at -20°C. For paraffin sections, tissues were fixed in 4% paraformaldahyde overnight at 4°C and then washed in 1X PBS 3 times for 20 minutes each at 4°C; followed by rotation in 0.85% NaCl 3 times for 20 minutes each at 4°C; then rotation in a 1:1 ratio of 0.85%NaCl/100%EtOH for 1h at RT; and finally rotation in 70% EtOH overnight at 4°C. The tissues were then rotated in 85% EtOH for 1h at RT; followed by 95% EtOH for 1h at RT; then in 100% EtOH 3 times for 20 minutes each at RT; then in xylene 3 times for 20 minutes each at RT; and finally in a 1:1 ratio of paraffin/xylene for 1.5h at 60°C; then paraffin for 45 min. at 60°C with vacuum; and finally paraffin for 45 mins with vacuum again. Tissue were then sectioned in 8–12 μm sections using Motorized Rotary Microtome (Leica), mounted on glass slides and stored in 4°C.

### In situ hybridization (ISH) for frozen sections

In situ hybridization on frozen sections was performed using 5’ DIG-labeled LNA probes (Exiqon) for miRNAs according to published protocols[23,74,75]. Briefly, tissue slides were first warmed and then washed with DEPC-treated 1X PBS 3 times for 3 minutes each, followed by acetylating in 100mM triethanolamine buffer plus 0.25% of acetic anhydride for 10 minutes, permeabilized in PBST (1X PBS plus 0.1% Triton X-100 in DEPC-treated water) for 30 minutes, and washed 3 times for 5 minutes each at RT in 1X PBS. After pre-hybridization (50% formamide, 10mM Tris-HCl pH8.0, 600mM NaCl, 1X Denhardt’s solution, 200μg/mL tRNA, 1mM EDTA, 0.25% SDS, 10% dextran sulfate) at RT for 2 hrs, hybridization was carried out at 50°C overnight in the same hybridization buffer containing 25nM of miR-29c (TAGCACCATTTGAAATCGGTTA) or miR-29a (TAGCACCATCTGAAATCGGTTA) and miR-29b (TAGCACCATTTGAAATCAGTGTT) DIG-labeled LNA probes. Then, slides were sequentially washed with SSC (2X, 1X, 0.2X) at 50°C for 30 min. followed by 0.2X SSC for 5 minutes at RT, and finally 1X PBS for 5 minutes at RT. Slides were then incubated in blocking solution (TTBS, 0.05M Tris, pH 7.5, 0.15M NaCl, 0.1% Tween-20, plus 5% sheep serum) and incubated with Anti-Digoxigenin-AP, Fab fragments (1:500, Roche, Cat.#11093274910) overnight at 4°C. After washing in TTBS 3 times for 10 minutes each, signals were developed using BM purple (Roche, Cat.# 11442074001).

### Immunofluorescence (IF) and immunohistochemistry (IHC)

For IHC, frozen sections were washed with 1X PBS three times followed by incubating with 0.3% H_2_O_2_/0.3% horse serum in 1X PBS for 5 minutes at RT. Then, slides were washed in TNT buffer (0.1M Tris pH 7.5, 0.15M NaCl, 0.05% Tween-20) 3 times. Slides were then blocked one hour with TNB buffer (0.1M Tris pH 7.5, 0.15M NaCl, 0.5% blocking reagent) at RT, followed by incubation with primary antibody (COL1A1, Rockland, 1:1000) diluted in TNB buffer overnight at 4°C. Slides were then washed with TNT buffer 3 times, incubated with Horseradish peroxidase (HRP) conjugated secondary antibodies (Life Technologies) for 2 hours at RT, signals were developed by using the DAB staining kit (Vector Laboratories, Cat.# SK-4100). For IF staining, frozen sections were washed in PBS, and then blocked by 5% donkey serum in PBST for one hour at RT, followed by incubation with primary antibodies (α-SMA, 1:3000; COL1A1, Rockland, 1:1000; KLF4, Cell Signaling Technology, 1:50; FBXO32, 1:100, ECM Bioscience; PDGFRB, 1:50, Cell Signaling Technology) overnight at 4°C. Alexa Fluor 488 or 568-conjugated secondary antibodies (Donkey anti-Mouse IgG, or Donkey anti-Rabbit IgG or Donkey anti-Goat IgG; Life Technologies, 1:250 dilution) were used for the visualization of signals. Nikon DS Ri1 and SPOT software were used for the image acquisition and analysis.

### Statistical analysis

For quantitative analyses including qRT-PCR of miRNA or mRNAs, 3’UTR luciferase, results from three or four independent experiments were subjected to 2-tailed, unpaired Student’s t test. RNA samples from two independent experiments were subjected for NGS of small RNAs. Data are presented as the mean ± SEM, P < 0.05 was considered significant.

### Study approval

All animal studies were carried out with the approval of the Institutional Animal Care and Use Committee (IACUC) in both the Boston University Medical Campus and Columbia University Medical Campus. Mice were housed in the Laboratory Animal Science Center (LASC), an approved animal facility, and received regular veterinary care and husbandry.

## Supporting Information

S1 Fig Levels of COL1A1, α-SMA and KLF4 are not significantly altered in SMCs of dorsal aorta. **(A)** in situ hybridization of miR-29c in dorsal aorta of adult mouse, followed by IF staining of α-SMA**(B). (C&D)** COL1A1 IF staining of WT and miR-29 DKO aortic walls. **(E&F)** Double IF staining of KLF4 and α-SMA in the aortic walls of WT and miR-29 DKO mice. Scale bar: 10 μM.
(TIF)

S2 Fig Levels of COL1A1, α-SMA and KLF4 are not significantly altered in airway SMCs of miR-29 DKO lungs. **(A&B)** Upregulation of COL1A1 in distal vessel walls were observed (white arrow) in DKO lungs, while levels of COL1A1 of airway SMCs is not significantly altered (yellow arrow). **(C&D)** Levels of α-SMA and KLF4 in airway SMCs are not significantly changed in DKO lungs (yellow arrow). Scale bar: 50μM.
(TIF)

S3 Fig Upregulation of miR-29 expression during postnatal lung development. Levels of miR-29 a/b/c in RNA samples of mouse lungs at different stages of postnatal development (qRT-PCR, n = 3). P<0.05 for all comparisons of E19 vs PND14, or E19 vs PND21 or E19 vs Adult.
(TIF)

S4 Fig No significant upregulation of FBXO32 in airway SMCs and vSMCs of large proximal vessels. **(A&B)** Double IF staining of FBXO32 (red) and α-SMA (green) in WT control and miR-29 DKO lungs. vSMCs of large proximal vessels (white arrow), and airway SMCs (yellow arrow). Scale bar: 100μM
(TIF)

S5 Fig miR-29 DKO mice develop smaller hearts. **A)** Hearts of miR-29 DKO and WT littermates at age four weeks were fixed overnight in 4% paraformaldehyde, paraffin-embedded, sectioned, and stained with H&E. **B)** HW, heart weight; LV, left ventricular weight; RV, right ventricular weight; TL, Tibial length were determined (n = 6 for each group), *P<0.05. **C)** HW, heart weight and BW, body weight were determined (n = 6 for each group), NS, no significant difference.
(TIF)

S1 Table Smooth muscle related genes reduced in miR-29 knockdown IMR-90 cells. (TIF)

S2 Table List of top ten most abundant miRNAs in adult mouse lungs. (TIF)

## References

[1] Stenmark KR, Abman SH (2005) Lung vascular development: implications for the pathogenesis of bronchopulmonary dysplasia. Annu Rev Physiol 67: 623–661. 15709973 10.1146/annurev.physiol.67.040403.102229

[2] Owens GK (2007) Molecular control of vascular smooth muscle cell differentiation and phenotypic plasticity. Novartis Found Symp 283: 174–191; discussion 191–173, 238–141. 18300422 10.1002/9780470319413.ch14

[3] Haworth SG (1995) Development of the normal and hypertensive pulmonary vasculature. Exp Physiol 80: 843–853. 8546873 10.1113/expphysiol.1995.sp003892

[4] Hall SM, Gorenflo M, Reader J, Lawson D, Haworth SG (2000) Neonatal pulmonary hypertension prevents reorganisation of the pulmonary arterial smooth muscle cytoskeleton after birth. J Anat 196 (Pt 3): 391–403. 10853961 10.1046/j.1469-7580.2000.19630391.x PMC1468075

[5] Zhou W, Dasgupta C, Negash S, Raj JU (2007) Modulation of pulmonary vascular smooth muscle cell phenotype in hypoxia: role of cGMP-dependent protein kinase. Am J Physiol Lung Cell Mol Physiol 292: L1459–1466. 17322285 10.1152/ajplung.00143.2006

[6] Morrell NW, Adnot S, Archer SL, Dupuis J, Jones PL, et al (2009) Cellular and molecular basis of pulmonary arterial hypertension. J Am Coll Cardiol 54: S20–31. 10.1016/j.jacc.2009.04.018 19555855 PMC2790324

[7] Tuder RM, Stacher E, Robinson J, Kumar R, Graham BB (2013) Pathology of pulmonary hypertension. Clin Chest Med 34: 639–650. 10.1016/j.ccm.2013.08.009 24267295

[8] Tuder RM, Abman SH, Braun T, Capron F, Stevens T, et al (2009) Development and pathology of pulmonary hypertension. J Am Coll Cardiol 54: S3–9. 10.1016/j.jacc.2009.04.009 19555856

[9] Farber HW, Loscalzo J (2004) Pulmonary arterial hypertension. N Engl J Med 351: 1655–1665. 15483284 10.1056/NEJMra035488

[10] Suzuki T, Aizawa K, Matsumura T, Nagai R (2005) Vascular implications of the Kruppel-like family of transcription factors. Arterioscler Thromb Vasc Biol 25: 1135–1141. 15817882 10.1161/01.ATV.0000165656.65359.23

[11] Wang DZ, Olson EN (2004) Control of smooth muscle development by the myocardin family of transcriptional coactivators. Curr Opin Genet Dev 14: 558–566. 15380248 10.1016/j.gde.2004.08.003

[12] Hughes AD, Clunn GF, Refson J, Demoliou-Mason C (1996) Platelet-derived growth factor (PDGF): actions and mechanisms in vascular smooth muscle. Gen Pharmacol 27: 1079–1089. 8981052 10.1016/s0306-3623(96)00060-2

[13] Hellberg C, Ostman A, Heldin CH (2010) PDGF and vessel maturation. Recent Results Cancer Res 180: 103–114. 10.1007/978-3-540-78281-0_7 20033380

[14] Rabinovitch M (2012) Molecular pathogenesis of pulmonary arterial hypertension. J Clin Invest 122: 4306–4313. 10.1172/JCI60658 23202738 PMC3533531

[15] West J (2010) Cross talk between Smad, MAPK, and actin in the etiology of pulmonary arterial hypertension. Adv Exp Med Biol 661: 265–278. 10.1007/978-1-60761-500-2_17 20204736

[16] Lagna G, Nguyen PH, Ni W, Hata A (2006) BMP-dependent activation of caspase-9 and caspase-8 mediates apoptosis in pulmonary artery smooth muscle cells. Am J Physiol Lung Cell Mol Physiol 291: L1059–1067. 17030903 10.1152/ajplung.00180.2006

[17] Bartel DP (2009) MicroRNAs: target recognition and regulatory functions. Cell 136: 215–233. 10.1016/j.cell.2009.01.002 19167326 PMC3794896

[18] Albinsson S, Skoura A, Yu J, DiLorenzo A, Fernandez-Hernando C, et al (2011) Smooth muscle miRNAs are critical for post-natal regulation of blood pressure and vascular function. PLoS One 6: e18869 10.1371/journal.pone.0018869 21526127 PMC3081311

[19] Albinsson S, Suarez Y, Skoura A, Offermanns S, Miano JM, et al (2010) MicroRNAs are necessary for vascular smooth muscle growth, differentiation, and function. Arterioscler Thromb Vasc Biol 30: 1118–1126. 10.1161/ATVBAHA.109.200873 20378849 PMC2880481

[20] Xin M, Small EM, Sutherland LB, Qi X, McAnally J, et al (2009) MicroRNAs miR-143 and miR-145 modulate cytoskeletal dynamics and responsiveness of smooth muscle cells to injury. Genes Dev 23: 2166–2178. 10.1101/gad.1842409 19720868 PMC2751981

[21] Paez-Cortez J, Krishnan R, Arno A, Aven L, Ram-Mohan S, et al (2013) A new approach for the study of lung smooth muscle phenotypes and its application in a murine model of allergic airway inflammation. PLoS One 8: e74469 10.1371/journal.pone.0074469 24040256 PMC3767675

[22] van Rooij E, Sutherland LB, Thatcher JE, DiMaio JM, Naseem RH, et al (2008) Dysregulation of microRNAs after myocardial infarction reveals a role of miR-29 in cardiac fibrosis. Proc Natl Acad Sci U S A 105: 13027–13032. 10.1073/pnas.0805038105 18723672 PMC2529064

[23] Cushing L, Kuang PP, Qian J, Shao F, Wu J, et al (2011) miR-29 is a major regulator of genes associated with pulmonary fibrosis. Am J Respir Cell Mol Biol 45: 287–294. 10.1165/rcmb.2010-0323OC 20971881 PMC3175558

[24] Ehler E, Babiychuk E, Draeger A (1996) Human foetal lung (IMR-90) cells: myofibroblasts with smooth muscle-like contractile properties. Cell Motil Cytoskeleton 34: 288–298. 8871816 10.1002/(SICI)1097-0169(1996)34:4<288::AID-CM4>3.0.CO;2-4

[25] Liu Y, Sinha S, McDonald OG, Shang Y, Hoofnagle MH, et al (2005) Kruppel-like factor 4 abrogates myocardin-induced activation of smooth muscle gene expression. J Biol Chem 280: 9719–9727. 15623517 10.1074/jbc.M412862200

[26] Chen CN, Li YS, Yeh YT, Lee PL, Usami S, et al (2006) Synergistic roles of platelet-derived growth factor-BB and interleukin-1beta in phenotypic modulation of human aortic smooth muscle cells. Proc Natl Acad Sci U S A 103: 2665–2670. 16477012 10.1073/pnas.0510973103 PMC1413813

[27] Stamatiou R, Paraskeva E, Gourgoulianis K, Molyvdas PA, Hatziefthimiou A (2012) Cytokines and growth factors promote airway smooth muscle cell proliferation. ISRN Inflamm 2012: 731472 10.5402/2012/731472 24049651 PMC3767366

[28] Kawai-Kowase K, Owens GK (2007) Multiple repressor pathways contribute to phenotypic switching of vascular smooth muscle cells. Am J Physiol Cell Physiol 292: C59–69. 16956962 10.1152/ajpcell.00394.2006

[29] Maurer B, Stanczyk J, Jungel A, Akhmetshina A, Trenkmann M, et al (2010) MicroRNA-29, a key regulator of collagen expression in systemic sclerosis. Arthritis Rheum 62: 1733–1743. 10.1002/art.27443 20201077

[30] Sekiya Y, Ogawa T, Yoshizato K, Ikeda K, Kawada N (2011) Suppression of hepatic stellate cell activation by microRNA-29b. Biochem Biophys Res Commun 412: 74–79. 10.1016/j.bbrc.2011.07.041 21798245

[31] Kwiecinski M, Elfimova N, Noetel A, Tox U, Steffen HM, et al (2012) Expression of platelet-derived growth factor-C and insulin-like growth factor I in hepatic stellate cells is inhibited by miR-29. Lab Invest 92: 978–987. 10.1038/labinvest.2012.70 22565577

[32] Talasila A, Yu H, Ackers-Johnson M, Bot M, van Berkel T, et al (2013) Myocardin regulates vascular response to injury through miR-24/-29a and platelet-derived growth factor receptor-beta. Arterioscler Thromb Vasc Biol 33: 2355–2365. 10.1161/ATVBAHA.112.301000 23825366

[33] Glass DJ (2003) Signalling pathways that mediate skeletal muscle hypertrophy and atrophy. Nat Cell Biol 5: 87–90. 12563267 10.1038/ncb0203-87

[34] Bodine SC, Baehr LM (2014) Skeletal muscle atrophy and the E3 ubiquitin ligases MuRF1 and MAFbx/atrogin-1. Am J Physiol Endocrinol Metab 307: E469–484. 10.1152/ajpendo.00204.2014 25096180 PMC4166716

[35] Lu T, Chai Q, Yu L, d'Uscio LV, Katusic ZS, et al (2012) Reactive oxygen species signaling facilitates FOXO-3a/FBXO-dependent vascular BK channel beta1 subunit degradation in diabetic mice. Diabetes 61: 1860–1868. 10.2337/db11-1658 22586590 PMC3379647

[36] Yi F, Wang H, Chai Q, Wang X, Shen WK, et al (2014) Regulation of large conductance Ca2+-activated K+ (BK) channel beta1 subunit expression by muscle RING finger protein 1 in diabetic vessels. J Biol Chem 289: 10853–10864. 10.1074/jbc.M113.520940 24570002 PMC4036198

[37] Zhang DM, He T, Katusic ZS, Lee HC, Lu T (2010) Muscle-specific f-box only proteins facilitate bk channel beta(1) subunit downregulation in vascular smooth muscle cells of diabetes mellitus. Circ Res 107: 1454–1459. 10.1161/CIRCRESAHA.110.228361 20966391 PMC3076051

[38] Tucka J, Yu H, Gray K, Figg N, Maguire J, et al (2014) Akt1 regulates vascular smooth muscle cell apoptosis through FoxO3a and Apaf1 and protects against arterial remodeling and atherosclerosis. Arterioscler Thromb Vasc Biol 34: 2421–2428. 10.1161/ATVBAHA.114.304284 25234814

[39] Lee HY, Chung JW, Youn SW, Kim JY, Park KW, et al (2007) Forkhead transcription factor FOXO3a is a negative regulator of angiogenic immediate early gene CYR61, leading to inhibition of vascular smooth muscle cell proliferation and neointimal hyperplasia. Circ Res 100: 372–380. 17234971 10.1161/01.RES.0000257945.97958.77

[40] Allard D, Figg N, Bennett MR, Littlewood TD (2008) Akt regulates the survival of vascular smooth muscle cells via inhibition of FoxO3a and GSK3. J Biol Chem 283: 19739–19747. 10.1074/jbc.M710098200 18458087

[41] Guerit D, Brondello JM, Chuchana P, Philipot D, Toupet K, et al (2014) FOXO3A regulation by miRNA-29a Controls chondrogenic differentiation of mesenchymal stem cells and cartilage formation. Stem Cells Dev 23: 1195–1205. 10.1089/scd.2013.0463 24467486

[42] Liston A, Papadopoulou AS, Danso-Abeam D, Dooley J (2012) MicroRNA-29 in the adaptive immune system: setting the threshold. Cell Mol Life Sci 69: 3533–3541. 10.1007/s00018-012-1124-0 22971773 PMC11114856

[43] He Y, Huang C, Lin X, Li J (2013) MicroRNA-29 family, a crucial therapeutic target for fibrosis diseases. Biochimie 95: 1355–1359. 10.1016/j.biochi.2013.03.010 23542596

[44] Wang Y, Zhang X, Li H, Yu J, Ren X (2013) The role of miRNA-29 family in cancer. Eur J Cell Biol 92: 123–128. 10.1016/j.ejcb.2012.11.004 23357522

[45] Pekarsky Y, Croce CM (2010) Is miR-29 an oncogene or tumor suppressor in CLL? Oncotarget 1: 224–227. 20936047 10.18632/oncotarget.129 PMC2951328

[46] Montgomery RL, Yu G, Latimer PA, Stack C, Robinson K, et al (2014) MicroRNA mimicry blocks pulmonary fibrosis. EMBO Mol Med 6: 1347–1356. 10.15252/emmm.201303604 25239947 PMC4287936

[47] Fabbri M, Garzon R, Cimmino A, Liu Z, Zanesi N, et al (2007) MicroRNA-29 family reverts aberrant methylation in lung cancer by targeting DNA methyltransferases 3A and 3B. Proc Natl Acad Sci U S A 104: 15805–15810. 17890317 10.1073/pnas.0707628104 PMC2000384

[48] Garzon R, Heaphy CE, Havelange V, Fabbri M, Volinia S, et al (2009) MicroRNA 29b functions in acute myeloid leukemia. Blood 114: 5331–5341. 10.1182/blood-2009-03-211938 19850741 PMC2796138

[49] Kogure T, Costinean S, Yan I, Braconi C, Croce C, et al (2012) Hepatic miR-29ab1 expression modulates chronic hepatic injury. J Cell Mol Med 16: 2647–2654. 10.1111/j.1582-4934.2012.01578.x 22469499 PMC3923513

[50] Li Z, Hassan MQ, Jafferji M, Aqeilan RI, Garzon R, et al (2009) Biological functions of miR-29b contribute to positive regulation of osteoblast differentiation. J Biol Chem 284: 15676–15684. 10.1074/jbc.M809787200 19342382 PMC2708864

[51] Pekarsky Y, Santanam U, Cimmino A, Palamarchuk A, Efanov A, et al (2006) Tcl1 expression in chronic lymphocytic leukemia is regulated by miR-29 and miR-181. Cancer Res 66: 11590–11593. 17178851 10.1158/0008-5472.CAN-06-3613

[52] Santanam U, Zanesi N, Efanov A, Costinean S, Palamarchuk A, et al (2010) Chronic lymphocytic leukemia modeled in mouse by targeted miR-29 expression. Proc Natl Acad Sci U S A 107: 12210–12215. 10.1073/pnas.1007186107 20566844 PMC2901490

[53] Smith KM, Guerau-de-Arellano M, Costinean S, Williams JL, Bottoni A, et al (2012) miR-29ab1 deficiency identifies a negative feedback loop controlling Th1 bias that is dysregulated in multiple sclerosis. J Immunol 189: 1567–1576. 10.4049/jimmunol.1103171 22772450 PMC3411895

[54] Wang H, Garzon R, Sun H, Ladner KJ, Singh R, et al (2008) NF-kappaB-YY1-miR-29 regulatory circuitry in skeletal myogenesis and rhabdomyosarcoma. Cancer Cell 14: 369–381. 10.1016/j.ccr.2008.10.006 18977326 PMC3829205

[55] Ott CE, Grunhagen J, Jager M, Horbelt D, Schwill S, et al (2011) MicroRNAs differentially expressed in postnatal aortic development downregulate elastin via 3' UTR and coding-sequence binding sites. PLoS One 6: e16250 10.1371/journal.pone.0016250 21305018 PMC3031556

[56] Wei W, He HB, Zhang WY, Zhang HX, Bai JB, et al (2013) miR-29 targets Akt3 to reduce proliferation and facilitate differentiation of myoblasts in skeletal muscle development. Cell Death Dis 4: e668 10.1038/cddis.2013.184 23764849 PMC3698551

[57] Ma W, Xie S, Ni M, Huang X, Hu S, et al (2012) MicroRNA-29a inhibited epididymal epithelial cell proliferation by targeting nuclear autoantigenic sperm protein (NASP). J Biol Chem 287: 10189–10199. 10.1074/jbc.M111.303636 22194605 PMC3323015

[58] Podolska A, Kaczkowski B, Kamp Busk P, Sokilde R, Litman T, et al (2011) MicroRNA expression profiling of the porcine developing brain. PLoS One 6: e14494 10.1371/journal.pone.0014494 21253018 PMC3017054

[59] Li Y, Piatigorsky J (2009) Targeted deletion of Dicer disrupts lens morphogenesis, corneal epithelium stratification, and whole eye development. Dev Dyn 238: 2388–2400. 10.1002/dvdy.22056 19681134 PMC2787093

[60] Wang L, Zhou L, Jiang P, Lu L, Chen X, et al (2012) Loss of miR-29 in myoblasts contributes to dystrophic muscle pathogenesis. Mol Ther 20: 1222–1233. 10.1038/mt.2012.35 22434133 PMC3369280

[61] Boettger T, Beetz N, Kostin S, Schneider J, Kruger M, et al (2009) Acquisition of the contractile phenotype by murine arterial smooth muscle cells depends on the Mir143/145 gene cluster. J Clin Invest 119: 2634–2647. 10.1172/JCI38864 19690389 PMC2735940

[62] Chen Z, Wu J, Yang C, Fan P, Balazs L, et al (2012) DiGeorge syndrome critical region 8 (DGCR8) protein-mediated microRNA biogenesis is essential for vascular smooth muscle cell development in mice. J Biol Chem 287: 19018–19028. 10.1074/jbc.M112.351791 22511778 PMC3365935

[63] Fan P, Chen Z, Tian P, Liu W, Jiao Y, et al (2013) miRNA biogenesis enzyme Drosha is required for vascular smooth muscle cell survival. PLoS One 8: e60888 10.1371/journal.pone.0060888 23637774 PMC3630177

[64] Elia L, Quintavalle M, Zhang J, Contu R, Cossu L, et al (2009) The knockout of miR-143 and -145 alters smooth muscle cell maintenance and vascular homeostasis in mice: correlates with human disease. Cell Death Differ 16: 1590–1598. 10.1038/cdd.2009.153 19816508 PMC3014107

[65] Cittelly DM, Finlay-Schultz J, Howe EN, Spoelstra NS, Axlund SD, et al (2013) Progestin suppression of miR-29 potentiates dedifferentiation of breast cancer cells via KLF4. Oncogene 32: 2555–2564. 10.1038/onc.2012.275 22751119 PMC4236860

[66] Tang W, Zhu Y, Gao J, Fu J, Liu C, et al (2014) MicroRNA-29a promotes colorectal cancer metastasis by regulating matrix metalloproteinase 2 and E-cadherin via KLF4. Br J Cancer 110: 450–458. 10.1038/bjc.2013.724 24281002 PMC3899762

[67] Turner EC, Huang CL, Govindarajan K, Caplice NM (2013) Identification of a Klf4-dependent upstream repressor region mediating transcriptional regulation of the myocardin gene in human smooth muscle cells. Biochim Biophys Acta 1829: 1191–1201. 10.1016/j.bbagrm.2013.09.002 24060351

[68] Cushing L, Kuang P, Lu J (2015) The role of miR-29 in pulmonary fibrosis. Biochem Cell Biol 93: 109–118. 10.1139/bcb-2014-0095 25454218

[69] Caruso P, Dempsie Y, Stevens HC, McDonald RA, Long L, et al (2012) A role for miR-145 in pulmonary arterial hypertension: evidence from mouse models and patient samples. Circ Res 111: 290–300. 10.1161/CIRCRESAHA.112.267591 22715469

[70] Wohrley JD, Frid MG, Moiseeva EP, Orton EC, Belknap JK, et al (1995) Hypoxia selectively induces proliferation in a specific subpopulation of smooth muscle cells in the bovine neonatal pulmonary arterial media. J Clin Invest 96: 273–281. 7615796 10.1172/JCI118031 PMC185198

[71] Prosser IW, Stenmark KR, Suthar M, Crouch EC, Mecham RP, et al (1989) Regional heterogeneity of elastin and collagen gene expression in intralobar arteries in response to hypoxic pulmonary hypertension as demonstrated by in situ hybridization. Am J Pathol 135: 1073–1088. 2596571 PMC1880484

[72] Stenmark KR, Orton EC, Reeves JT, Voelkel NF, Crouch EC, et al (1988) Vascular remodeling in neonatal pulmonary hypertension. Role of the smooth muscle cell. Chest 93: 127S–133S. 3342691

[73] Davis-Dusenbery BN, Chan MC, Reno KE, Weisman AS, Layne MD, et al (2011) down-regulation of Kruppel-like factor-4 (KLF4) by microRNA-143/145 is critical for modulation of vascular smooth muscle cell phenotype by transforming growth factor-beta and bone morphogenetic protein 4. J Biol Chem 286: 28097–28110. 10.1074/jbc.M111.236950 21673106 PMC3151055

[74] Mam V, Tanbe AF, Vitali SH, Arons E, Christou HA, et al (2010) Impaired vasoconstriction and nitric oxide-mediated relaxation in pulmonary arteries of hypoxia- and monocrotaline-induced pulmonary hypertensive rats. J Pharmacol Exp Ther 332: 455–462. 10.1124/jpet.109.160119 19915069 PMC2812110

[75] Qiao L, Xie L, Shi K, Zhou T, Hua Y, et al (2012) Notch signaling change in pulmonary vascular remodeling in rats with pulmonary hypertension and its implication for therapeutic intervention. PLoS One 7: e51514 10.1371/journal.pone.0051514 23251561 PMC3520790

