# Supplementary figures and images for: Disruption of miR-29 Leads to Aberrant Differentiation of Smooth Muscle Cells Selectively Associated with Distal Lung Vasculature
Source: PLoS Genet. 2015 May 28;11(5):e1005238. doi: 10.1371/journal.pgen.1005238 (PMC4447351; doi:10.1371/journal.pgen.1005238)

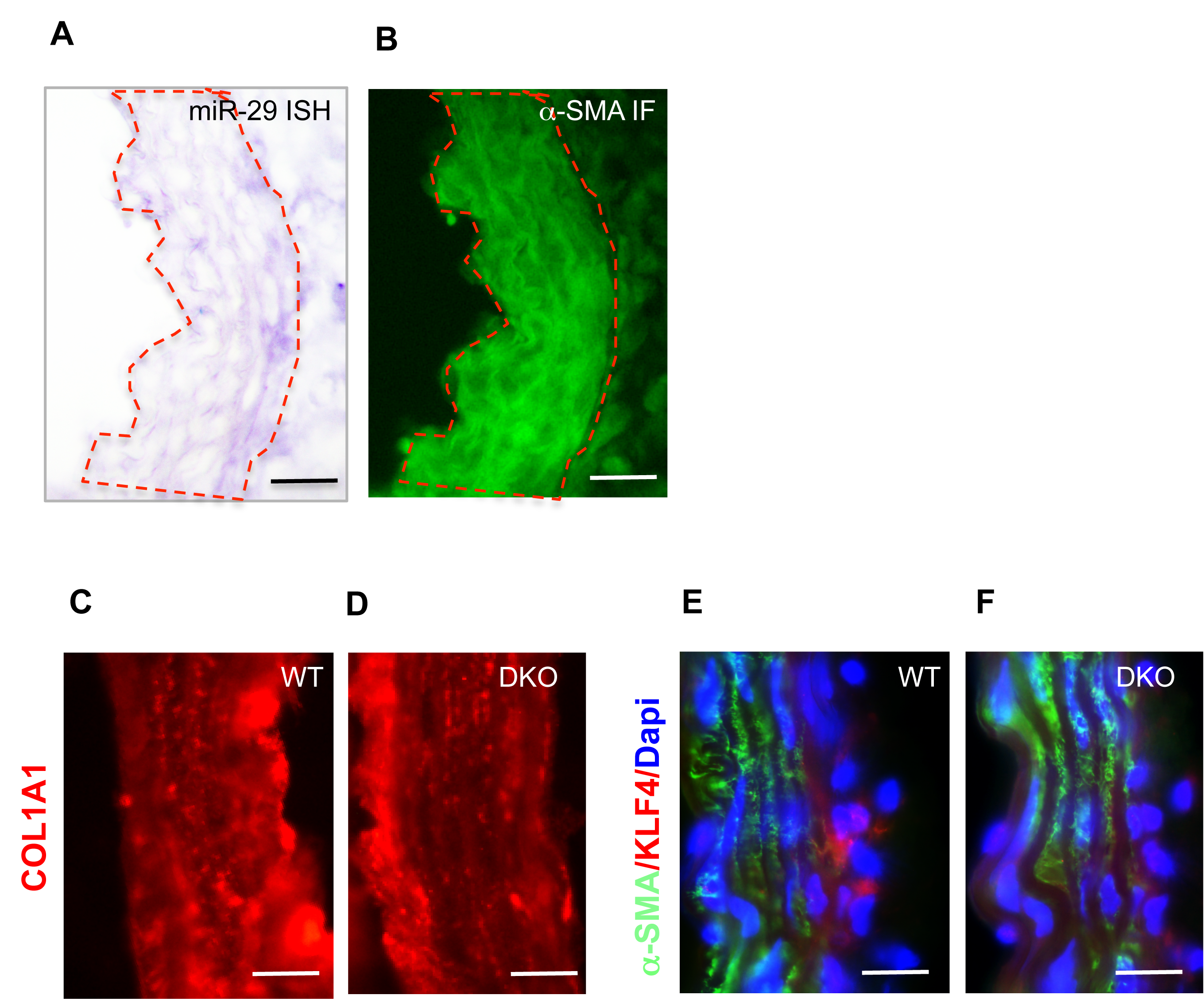

Supplement: S1 Fig — (A) in situ hybridization of miR-29c in dorsal aorta of adult mouse, followed by IF staining of α-SMA(B). (C&D) COL1A1 IF staining of WT and miR-29 DKO aortic walls. (E&F) Double IF staining of KLF4 and α-SMA in the aortic walls of WT and miR-29 DKO mice. Scale bar: 10 μM. (TIF) [file pgen.1005238.s001.tif]

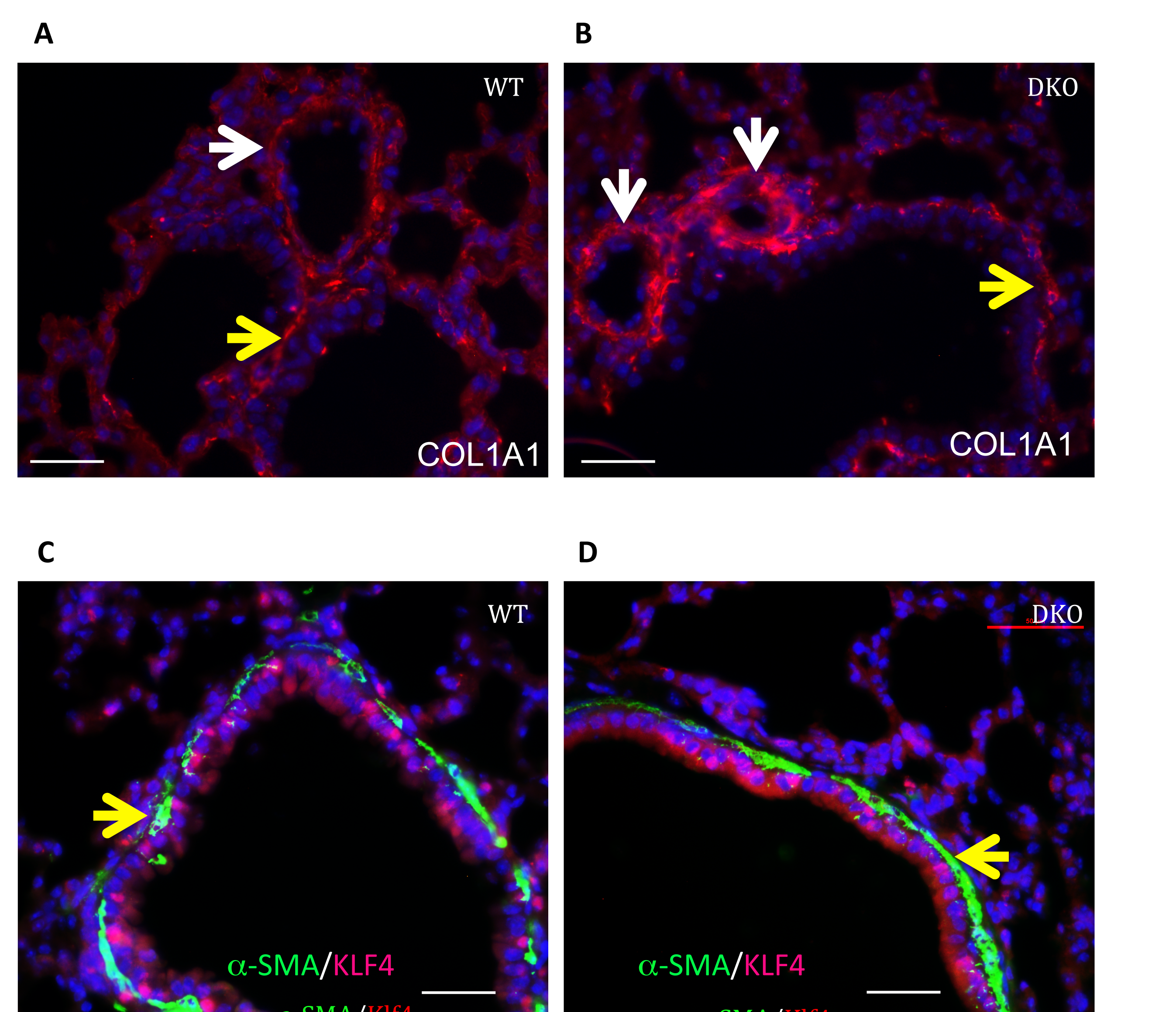

Supplement: S2 Fig — (A&B) Upregulation of COL1A1 in distal vessel walls were observed (white arrow) in DKO lungs, while levels of COL1A1 of airway SMCs is not significantly altered (yellow arrow). (C&D) Levels of α-SMA and KLF4 in airway SMCs are not significantly changed in DKO lungs (yellow arrow). Scale bar: 50μM. (TIF) [file pgen.1005238.s002.tif]

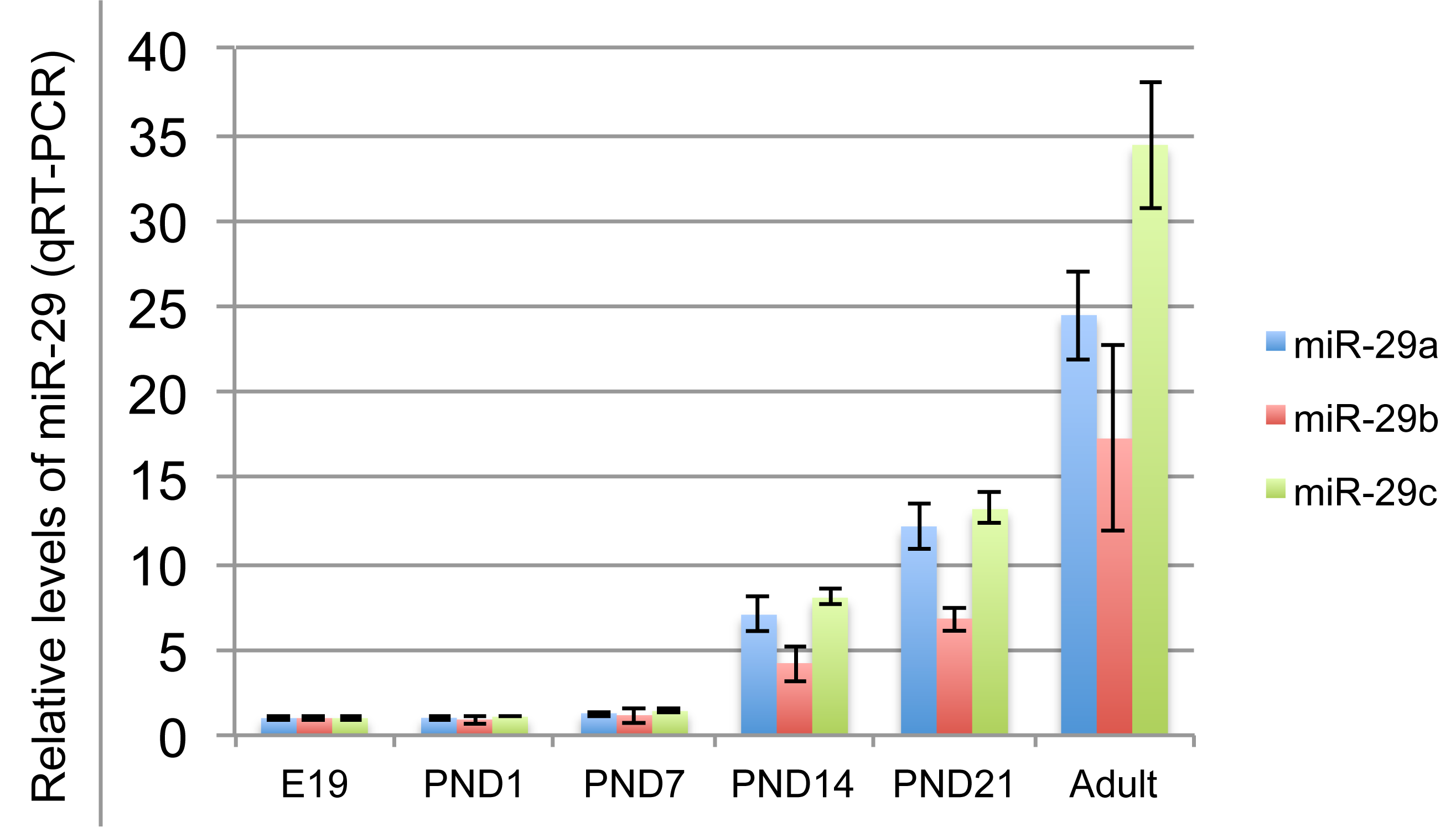

Supplement: S3 Fig — Levels of miR-29 a/b/c in RNA samples of mouse lungs at different stages of postnatal development (qRT-PCR, n = 3). P<0.05 for all comparisons of E19 vs PND14, or E19 vs PND21 or E19 vs Adult. (TIF) [file pgen.1005238.s003.tif]

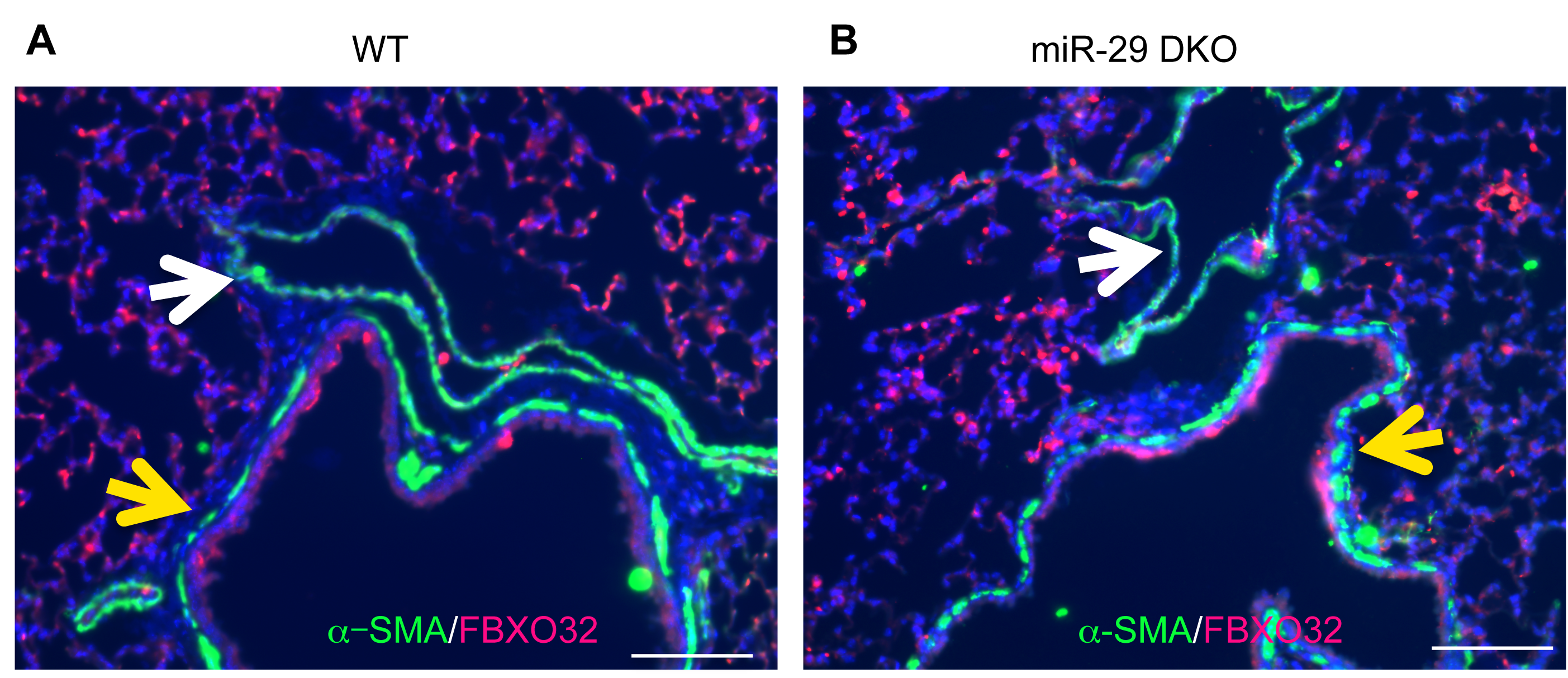

Supplement: S4 Fig — (A&B) Double IF staining of FBXO32 (red) and α-SMA (green) in WT control and miR-29 DKO lungs. vSMCs of large proximal vessels (white arrow), and airway SMCs (yellow arrow). Scale bar: 100μM (TIF) [file pgen.1005238.s004.tif]

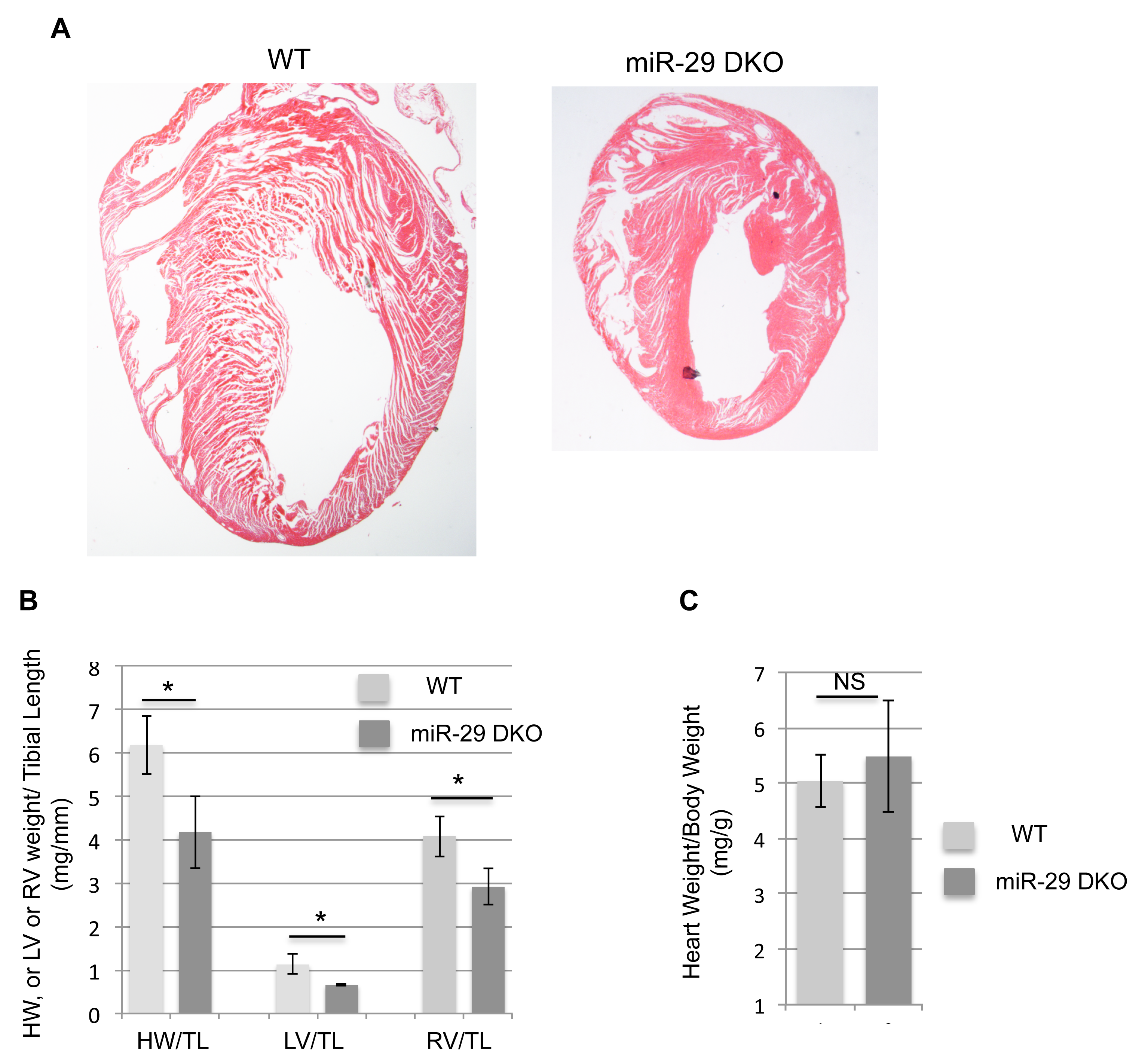

Supplement: S5 Fig — A) Hearts of miR-29 DKO and WT littermates at age four weeks were fixed overnight in 4% paraformaldehyde, paraffin-embedded, sectioned, and stained with H&E. B) HW, heart weight; LV, left ventricular weight; RV, right ventricular weight; TL, Tibial length were determined (n = 6 for each group), *P<0.05. C) HW, heart weight and BW, body weight were determined (n = 6 for each group), NS, no significant difference. (TIF) [file pgen.1005238.s005.tif]

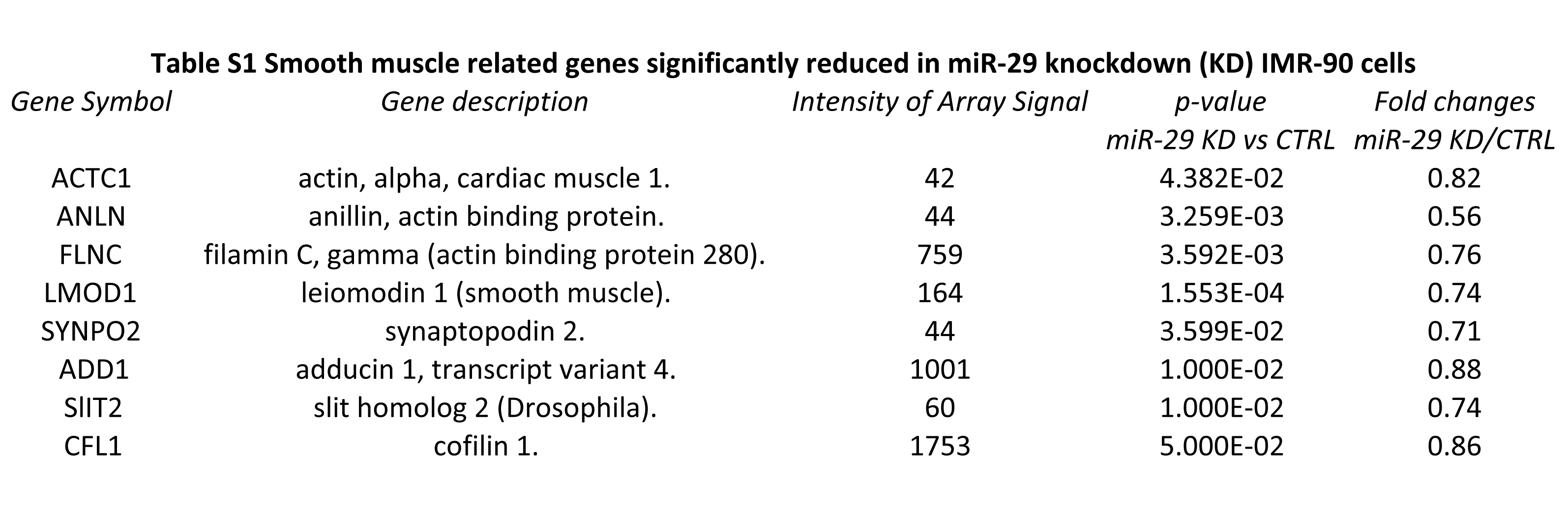

Supplement: S1 Table — (TIF) [file pgen.1005238.s006.tif]

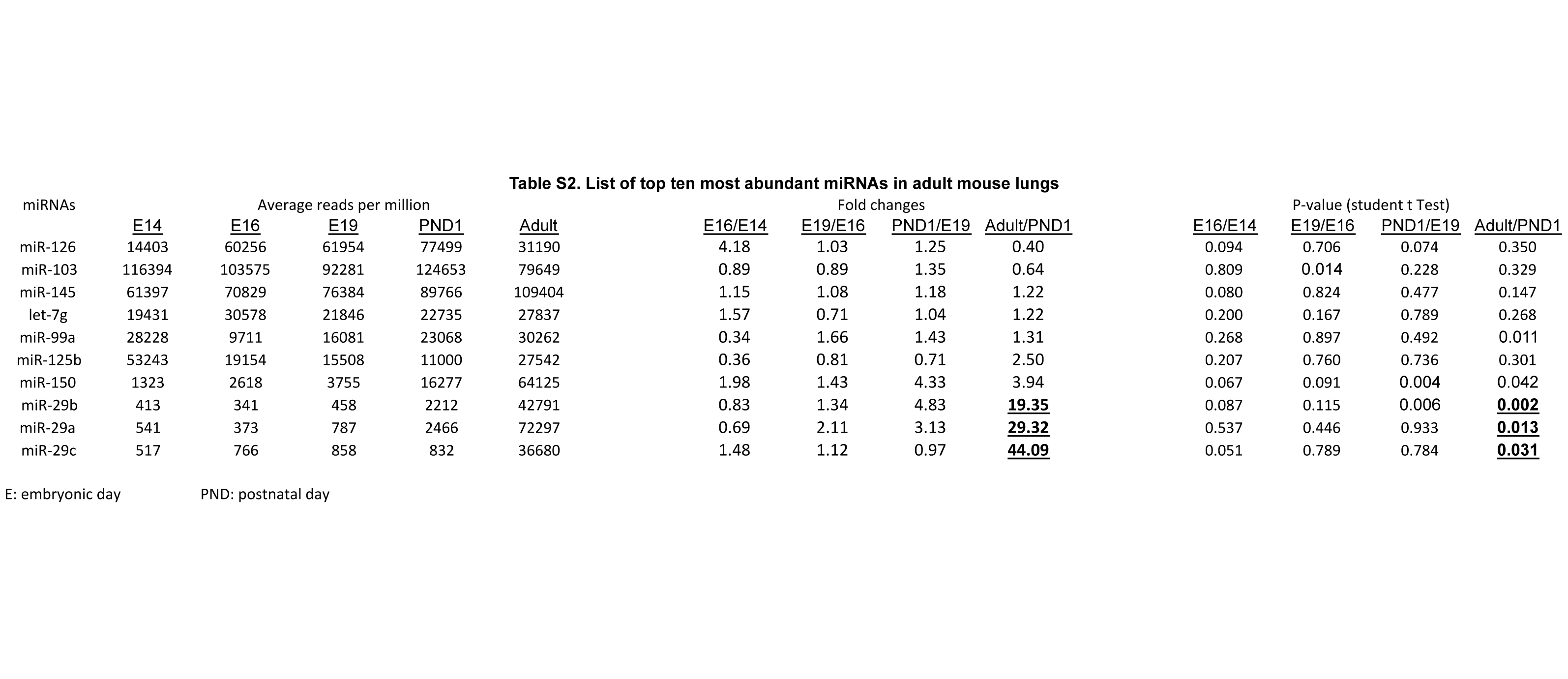

Supplement: S2 Table — (TIF) [file pgen.1005238.s007.tif]
